# Sustaining a nursing best practice guideline in an acute care setting over 10 years: A mixed methods case study

**DOI:** 10.3389/frhs.2022.940936

**Published:** 2022-08-30

**Authors:** Letitia Nadalin Penno, Ian D. Graham, Chantal Backman, Jessica Fuentes-Plough, Barbara Davies, Janet Squires

**Affiliations:** ^1^Faculty of Health Sciences, School of Nursing, University of Ottawa, Ottawa, ON, Canada; ^2^Faculty of Medicine, School of Epidemiology and Public Health, University of Ottawa, Ottawa, ON, Canada; ^3^Clinical Epidemiology Program, Ottawa Hospital Research Institute, Ottawa, ON, Canada; ^4^Business School and Leadership School, Anahuac-Mayab University, Mérida, Yucatan, Mexico

**Keywords:** sustainability, Best Practice Guidelines, evidence-based practices, quality improvements, nursing, interventions, innovations, pain

## Abstract

**Background:**

To improve patient outcomes many healthcare organizations have undertaken a number of steps to enhance the quality of care, including the use of evidence-based practices (EBPs) such as clinical practice guidelines. However, there is little empirical understanding of the longer-term use of guideline-based practices and how to ensure their ongoing use. The aim of this study was to identify the determinants and knowledge translation interventions (KTIs) influencing ongoing use of selected recommendations of an institutional pain policy and protocol over time from an organizational perspective and 10 years post implementation on two units within an acute care setting.

**Methods:**

We conducted a mixed methods case study guided by the Dynamic Sustainability Framework of an EBP 10 years post implementation. We examined protocol sustainability at the nursing department and unit levels of a multi-site tertiary center in Canada. Data sources included document review (*n* = 29), chart audits (*n* = 200), and semi-structured interviews with nurses at the department (*n* = 3) and unit (*n* = 16) level.

**Results:**

We identified 32 sustainability determinants and 29 KTIs influencing ongoing use of an EBP in acute care. Three determinants and eight KTIs had a continuous influence in all three time periods: implementation phase (0–2 yrs), sustained phase (>2–10 yrs.), and at the 10-year mark. Implementation of KTIs evolved with the level of application (e.g., department vs. unit) to fit the EBP within the context highlighting the need to focus on determinants influencing ongoing use. Sustainability was associated with continual efforts of monitoring and providing timely feedback regarding adherence to recommendations. KTIs used to embed recommendations into routine practices/processes positively influenced high adherence rates. Use of a participatory approach for implementation and sustainment and linking KTIs designed to incrementally address low adherence rates facilitated sustainment.

**Conclusion:**

This research provides insight into the relationship between implementation and sustainability determinants and related KTIs during implementation and sustained use phases. Unique determinants identified by department and unit nurses reflect their different perspectives toward the innovation based on their respective roles and responsibilities. KTIs fostered changed behaviors and facilitated EBP sustainment in acute care. Findings confirm the concept of sustainability is a dynamic “ongoing process.”

## Introduction

The sustainability of evidence-based practices (EBPs) in clinical practice remains the least understood aspect of the research translation process (1, 2). Sustaining hospital based innovations remains suboptimal (3) posing a significant challenge to hospital practitioners and researchers (3, 4). Specifically, Wiltsey-Stirman et al. (5) highlight partial sustained use of EBPs within most studies (64% or 80 out 125 studies) varies between 6 months to over 2 years following initial implementation. One of the key aspects underlying partial sustainability in healthcare is the nature of the complex ever-changing environments into which the EBPs are being integrated (6). Managing and supporting the adaption of an EBP, within a changing context (6, 7) implies it is never isolated from the context within which it is implemented, nor from the individuals it impacts. Studies have identified, in specific contexts key innovation (3, 5, 8–15), individual user (3, 14, 16, 17), contextual determinants (3, 18–20), and in some studies, specific leadership determinants influencing the sustained use of EBPs among nurses (3, 17, 21–28). To date, reviews also indicate sustainment of EBPs remains a persistent challenge across a range of healthcare settings (1, 2, 29–31) highlighting the need to examine the determinants influencing sustainability in specific healthcare contexts, such as acute care (1, 3). This is particularly important given governments and health agencies growing interest in expenditures within acute care settings. For example, Ontario Ministry of Health and Long Term Care (MOH and LTC) reports indicate expenditures remain the largest in tertiary settings (32) and sustained EBPs not only could improve the quality of patient care but potentially reduce costs. Due to the gap that exists in our understanding of the determinants and KTIs influencing sustainment of EBPs, there is a need to conduct studies aimed at uncovering the “complex and evolving nature of healthcare innovation sustainability” (33), especially in acute care settings over time.

In acute care, nurses are often faced with the challenge of assessing and intervening to manage people's pain as part of their nursing practice. Evidence demonstrates unrelieved or poorly managed pain is a burden on the person and health-related system throughout the world (34). It is estimated that ~19% of the population in industrial countries live with some form of pain (35). In Canada, pain is the most common reason health consumers seek assistance and accounts for up to 78% of presenting complaints in emergency departments (36). Reports further reveal the prevalence of persistent pain in 65% of older adults (>65 years of age) (34, 37), and inadequate management of pain remains across all age groups (38).

In 2007, to address an identified need for “consistent pain care,” the study site's Nursing department, partnered with Register Nurses Association of Ontario (RNAO) to implement nine Best Practice Guidelines (BPGs), referred internally as the BPG Implementation Program (BPG-IP). The RNAO's Pain Assessment and Management BPG (Pain BPG) (39) was used to develop an internal pain policy and protocol (Pain P/P). Unlike the other eight (out of nine) BPGs, the Pain P/P was uniquely implemented across all inpatient units. By 2016, in a research planning meeting, nursing leaders reported that despite early implementation success, internal monitoring had demonstrated inconsistent use of Pain P/P recommendations among the Medicine care units compared to other inpatient units. Inconsistencies highlighted the need to examine what the organization had done to sustain the use of the Pain P/P over time (2007–2017), and to uncover the factors and point of care processes/practices influencing Medicine care nurses' use of the Pain P/P 10 years post initial implementation (2017). Thus, to advance knowledge on the long-term sustainability of a nursing BPG we examined the ongoing use of the Pain P/P with the expectation it would have broad application to a variety of nursing environments.

The aim of this study was to understand from a nursing department and unit level, the determinants and knowledge translation interventions (KTIs) that influenced nurses' use of selected Pain P/P recommendations, over a 10-year period (e.g., 2007–2017), within a large, multi-site, academic, acute care center. The objectives included (i) identifying nurses' perceptions of the determinants influencing department and unit level nurses' use of the Pain P/P recommendations over time and 10 years post implementation, (ii) verifying unit nurses' Pain P/P use 10 years post-implementation, and (iii) identifying the related KTIs influencing Pain P/P use over time, and 10 years post-implementation. This system wide approach to identify determinants influencing adherence and the changes needed to address the sustained use of an EBP in practice aligned with the primary investigator's leadership experience in clinical administration (e.g., Chief Executive Officer, Academic Dean) and management (e.g., Chief Nursing Officer, Director Critical Care). It also aligned with coauthors' expertise in theory development and application, long term research programs, and practice changing implementation research.

## Methods

### Design

We conducted an explanatory mixed method case study (40, 41) in a multi-site, academic, acute care center to understand the complexity of sustainability in a natural, organizational setting (41) and to further explain quantitative results from a chart audit (40). Specifically, to address study objectives 1 and 3; we first reviewed all documents related to the initial implementation (0–2 yrs) and ongoing use of the Pain P/P over time (>2–10 yrs) followed by qualitative interviews of departmental level nurses to examine how the Pain P/P was sustained at the nursing department level over time (2007–2017). To address objective 2, we then conducted a chart audit 10 years post implementation (2017) to verify nurses documented adherence to selected Pain P/P recommendations on two Medicine care units (embedded subcases). This was followed by qualitative interviews of same to further explain audit findings and to address objectives 1 and 3 at the unit level. The reporting of this case study adheres to Mixed Methods Article Reporting Standards (MMARS) (42) (see Supplementary material 1).

### Setting and pain BPG recommendations

#### Setting

The setting was a large Canadian, urban, multi-site, academic, acute care center composed of three sites with ~50,860 patient admissions annually, more than 60 inpatient and outpatient units combined, 1,122 staffed beds and more than 4,500 nurses. The decision point to use the Pain P/P rested with nurses at the clinical practice level.

#### Pain BPG recommendations

In 2007, the Pain P/P was comprised of 8 recommendations (R) which was updated to include a ninth recommendation (R9) based on the 2013 RNAO Pain BPG (38) (see Table 1). Recommendations included: (R1)—assess pain on admission to the unit; (R2)—assess pain once per shift and during hourly rounding; (R3)—establish pain management goals; (R4)—collaborate with patients to establish interventions to manage pain; (R5)—evaluate patient outcomes and effectiveness of interventions; (R6)—consult with pain management experts as required; (R7)—educate patients about their pain management plan; (R8)—document pain goal and management plan; and (R9)—educate nursing staff and physicians on pain assessment and management. Recommendations 1 and 2 are outlined in the policy as required assessments. All remaining recommendations are dependent on patient need. For this study, we examined 5 of 9 Pain P/P recommendations (R1–R4, R7) based on the following reasons:(i) they can all be evaluated clinically using an objective measure (e.g., numeric rating, prescribed intervention, pain goal rating), (ii) they are all explicitly documented in specified locations within inpatient health records, and (iii) they are all supported by one of the highest levels of evidence (1b), namely at least one randomized control trial (38). Initially the hospital took advantage of several RNAO external KTIs designed to support implementation and build capacity at the individual, and organizational levels, such as Best Practice Spotlight Organization (BPSO) symposia, summer institutes, champion network events, and toolkit training. Post the implementation use phase, the site's Nursing Professional Practice (NPP) department lead the initiative with the assistance of nursing managers, educators and champions, To date, the NPP department goals within the hospital remain: to improve patient outcomes and the quality of nursing care. Similarly, nursing strategic objectives remain: to support the utilization of EBPs and the evaluation of nurse sensitive indicators hospital–wide.

**Table 1:** Pain P/P target behaviors, RNAO Pain Assessment and Management BPG (38, 43) recommendation and level of evidence (44).

**Site Pain P/P number**.	**Pain P/P target behavior**	**RNAO pain assessment and management BPG**
		**Recommendation Number Level of Evidence**
	**Selected recommendations under review**	
1	Screen inpatients for presence of pain on 1) Each initial contact/admission (2007 and 2013)	**Assessment** Recommendation - 1.1 Level of Evidence -Ib
2	Ongoing assessments of Pain using standardized tools 1) Once per shift (2007). 2) During hourly rounding (2013)	**Assessment** Recommendation - 1.2 Level of Evidence - Ib
3	Establish an individualized goal for pain management with the patient (2007 and 2013).	**Planning** Recommendation - 2.1 Level of Evidence – Ib
4	Collaborate with the patient in establishing an individualized strategy and interventions to manage the patient's pain based on the best evidence and available resources (2007 and 2013).	**Planning** Recommendation - 2.1 Level of Evidence – Ib
7	Educate patient and families about their individualized pain management plan (2007 and 2013).	**Implement** Recommendation - 3.3 Level of Evidence – Ib
**Recommendations not under review in this study**		
5	Assess effects of pharmacological interventions at peak effect following administration and on an ongoing basis (2007 and 2013).	**Implement** Recommendation - 3.1 Level of Evidence – IIb
6	Consult with pain management experts (interdisciplinary team members) as required (e.g., in complex situations, escalating or unrelieved pain after a reasonable trial of management) (2007 and 2013).	**Planning** Recommendation - 2.2 Level of Evidence- Ib
8	Ensure ongoing documentation reflects patient goals, pain mgmt. plan, assessment, response to treatment, outcomes, and communicate to inter professional team (2007, 2013)	**Evaluation** Recommendation - 4.4 Level of Evidence - IIb
9	Completion of self-learning training modules for nurses and physicians (2013)	**Education** Recommendation - 5.4 Level of Evidence - IV

Key: Level of Evidence

R, Recommendation; CA, C.hart Audit; Q, Question; mgmt., management; hxy, history; txmt, treatment.

Ia Evidence obtained from meta-analysis or systematic reviews of randomized controlled trials.

Ib Evidence obtained from at least one randomized controlled trial.

IIa Evidence obtained from at least one well-designed controlled study without randomization.

IIb Evidence obtained from at least one other type of well-designed quasi- experimental study, without randomization.

III Evidence obtained from well-designed non-experimental descriptive studies, such as comparative studies, correlation studies and case studies.

IV Evidence obtained from expert committee reports or opinions and/or clinical experiences of respected authorities.

### Data collection

We used the Dynamic Sustainability Framework's (DSF) (6) (see Supplementary material 2) to guide data collection, analysis, and to present results for the following three time periods: implementation phase (0–2 yrs.), sustained use phases at 2–10 year, and at 10 years. Documents and departmental nurses' responses' provided data for study objectives 1 (determinants), and 3 (KTIs) over time. Audits provided data for objective 2 (adherence rate to selected BPG recommendations) at the 10-year timeframe. Audits, documents and unit nurses' responses provided data for study objectives 1, 2, and 3 at the 10-year timeframe.

#### Document collection for the implementation (0–2 yrs) and sustained use phases (>2–10 yrs)

The period of study was 10 years (2007–2017). We collected data from 29 documents (i.e., seven reports, 20 internal and 2 external), spanning 2005–2017, related to the Pain P/P, to gain a historical perspective of the determinants and KTIs used to sustain the Pain P/P over time. All documents were provided by nursing administration and included in this study. Notably, a significant amount of work was done to prepare for the implementation phase, hence the inclusion of documents between 2005 and 2007.

#### Audit data collection for the 10 year timeframe

**Figure 1:**
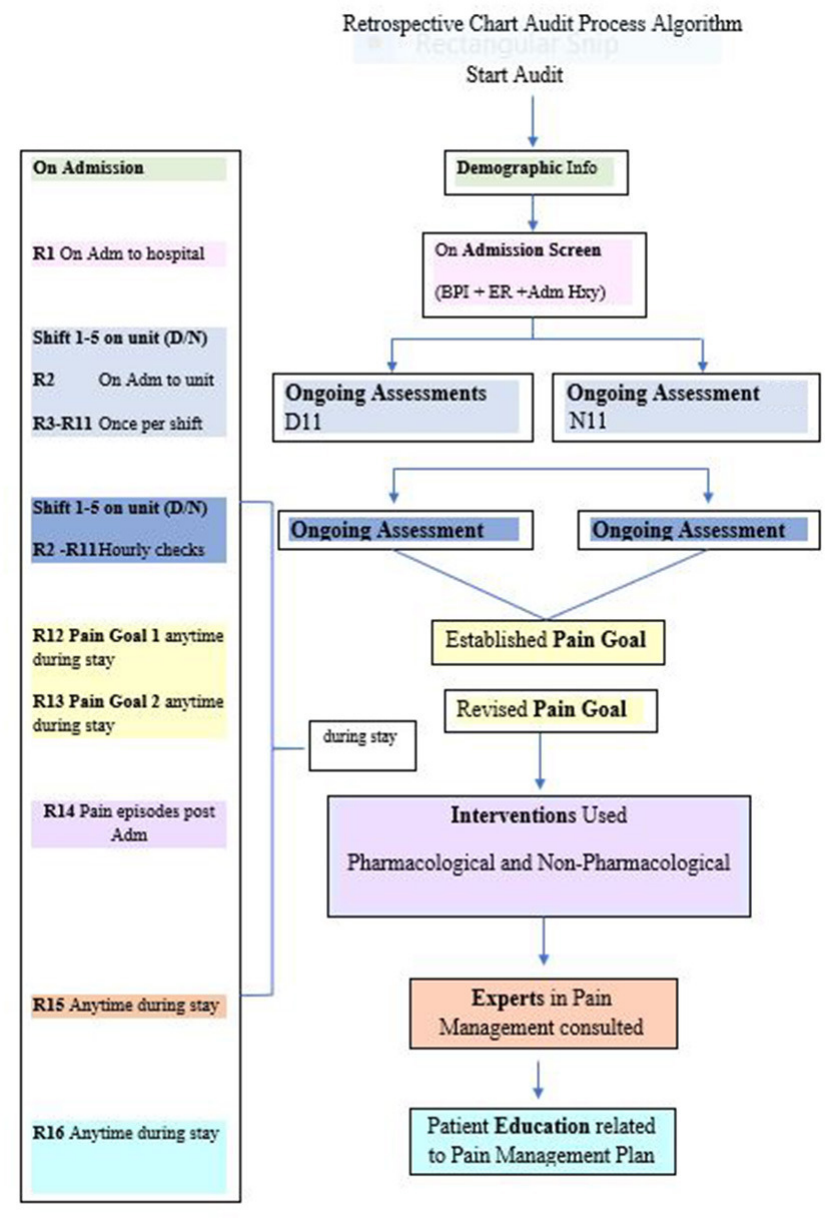
Retrospective chart audit process algorithm.

Organizational leaders purposefully selected two Medicine care units as “critical sub-cases” (41) among the existing five Medicine units. Subcase selection was based on maximum variation, managers' willingness to participate, biannual prevalence results, site uniqueness, and representation from different campuses. In early 2019, we conducted the chart audits for the selected subcases. We audited 100 randomly selected “unique” inpatient charts, for each subcase (total *n* = 200) to verify subcase nurses' adherence to five Pain P/P recommendations at the 10-year timeframe. We used the following audit dates, which were outside holiday periods, and proceeded established audit survey processes: August to October 2016, January to March 2017, and July to October 2017. The following audit tools were used: (i) process algorithm (see Figure 1), (ii) a coding dictionary, and (iii) an excel data extraction spreadsheet based on recommendation measures. Audit tools were subject to “face validity” testing (45) by two site representatives and one knowledge user (i.e., previous employee at study site) on the research team, then piloted. Two reviewers independently assessed the “reliability” of the extraction tool for 15 records (45) with minimal modifications to expand two data categories: patient admission diagnoses, alternative therapies used. For each recommendation, we specified inclusion criteria and sources. To maintain measurement consistency, we used the “first shift on the unit” as a measure of “*on admission*,” and the “next five consecutive shifts” as a measure for “*ongoing assessments*” (during patient stay). Post audit, an independent reviewer randomly tested extracted data calculations to confirm accuracy.

#### Interviews: Departmental and unit level

Semi-structured interview guides for department and unit nurses were developed based on DSF tenets (6) and the Pain P/P target behaviors. Pilot testing of interview guides (46) was undertaken with NPP representatives not selected for interviews. Only minor changes to the wording of the guide questions were made to ensure they were more open ended (available upon request).

In late 2018, we obtained REB approval. Based on similar studies (33, 47), we purposefully selected three department level participants who continued to be part of the implementation team over 10 years, and still available for interview. With the help of an internal gatekeeper, participants were emailed a study information letter and followed-up *via* phone and or email. All agreed to participate. Interviews were conducted in early 2019 *via* phone separately, each lasting 40–45 min. With consent, all interviews were digitally recorded and transcribe verbatim. Interviews were conducted sequentially by the researcher (LNP) and redundancy of responses was evident (46).

By late summer 2019 we obtained special permission from nursing administration to conduct unit interviews despite restrictions due to ongoing internal electronic health record changes. Managers on the selected units facilitated recruitment of nurses. Unit nurses were provided information regarding the study and were given allotted time to attend an interview while on duty with the researcher (LNP). Each unit consisted of ~30 full-time and several part time RNs. Participation was voluntary. Inclusion criteria for unit nurses included: full or part-time status, employed at least 2 years or more on the unit, and registered with the College of Nurses. Unit interviews were conducted between August and September 2019, completing one unit before moving onto the second. All interviews were held separately on each unit, lastly ~35–45 min. Interviews were recorded and transcribed verbatim. Based on similar studies (15), a convenient sample of eight to ten staff nurses per unit was planned. Interviews were conducted sequentially one unit at a time, until no new themes emerged, redundancy of responses was evident (46, 47).

### Analysis

#### Document analysis for the implementation (0–2 yrs) and sustained use phases (>2–10 yrs)

Initially, based on available data (i.e., 29 documents) we conducted a review of the changes that occurred over time. First we mapped the measures used in the biannual prevalence audit tools with the Pain P/P recommendations and education training records. Subsequently, a document review was conducted and a listing of KTIs (i.e., strategies) used over time (2005–2017), across all units to promote use of the recommendations was developed (see Supplementary material 3). We triangulated data sources with interview findings from departmental participants who worked across all units to clarify and enhance data completeness related to the determinants and KTIs influencing Pain P/P use. We aggregated all data findings to the nursing department level.

#### Audit data analysis for the 10 year timeframe

We analyzed audit data using descriptive statistical techniques using SPSS 25. Adherence rate calculations (i.e., the degree to which practitioners continued to adhere to guideline recommendations) (48, 49) involved determining indicators for targeted behaviors and computing frequency measurements. Adherence rates were calculated separately for each subcase. Findings were compared to adherence rate categories (high to low) consistent with previous studies (48, 49). Aggregated unit level adherence rates to guideline recommendations are described as high (80–100%), moderate (between 50 and 80%) or low (<50%) for each targeted behavior (48, 49). We compared differences in proportions between the subcases using Pearson's chi-square test for each of the recommendations (45). We used chart audit standards for quality set out by Gregory et al. (50). We triangulated findings with collated documents to validate the interpretation and inferences attained from the adherence rates (46).

#### Interview analysis for the three timeframes

Two independent reviewers conducted coding and interpretation of qualitative data, using content analysis (51). Interviews were digitally recorded, transcribed verbatim, and text were analyzed continuously until saturation. Specifically, we used NVivo 10 software to organized and facilitate coding of the data. Content analysis involved deductively separating and coding the interview responses and document data into groupings as per the DSF tenets (e.g., themes) (41), then inductively into smaller groupings (e.g., factors and related KTIs) (51) by timeline, by two independent reviewers (LNP and JF) for the department nurses. Similarly, the same analysis was conducted for the unit nurse responses. The few discrepancies were resolved through discussion and agreement. *Determinants* were considered factors that affected use of the Pain P/P, such as *barriers and facilitators*. *KTIs* were considered *strategies/action*s deliberately employed with the intention of promoting Pain P/P use. Within-in subcase descriptions, themes and summaries were analyzed separately, integrating all data sources. We analyzed themes “across subcases” for similarities and differences (41). In the final integration, we combined results for all three timeframes, and drew conclusions. Consistent with previous research (33), we used Lincoln and Guba's criteria (52) (credibility, dependability, confirmability, and transferability) for the qualitative portions to ensure rigor (see Table 2).

**Table 2:** Qualitative strategies for study rigor.

**Criterion**	**Strategies**
Credibility	• Used data from multiple sources, • Included multiple subcases, • Debriefing the research team, • Substantiated findings with participants during interviews
Dependability	• Adhering to study protocol, • Documenting decision points, • Maintaining organized databases, • Composing field notes, • Maintaining master lists of definitions and codes.
Confirmability	• Confirming eligibility, • Using the stopping criteria of three or more interviews where no new themes emerged as a measure of data redundancy (47), • Remaining close to verbatim transcripts, • Reviewed findings with knowledge users.
Transferability	• Providing detailed characteristics of the setting and participants, • Reported in-depth descriptions of findings • Used conceptual framework • Included critical subcases

### Ethics approval

Before commencement of data collection, ethical approval was obtained from the Research Ethics Boards for the site and the University of Ottawa. Organizational consent to examine ongoing use of the pain BPG was provided by all levels of nursing administration. Participation was voluntary. Participants signed a consent and completed a demographic form confirming eligibility prior to participation. We used unique identifiers to ensure anonymity of datasets and findings. Only aggregated data are reported. All quantitative extracted data were encrypted and password protected. The primary researcher and a site representative, maintained a table linking inpatient charts and coded reference numbers for each. Data remains stored in a secure location.

## Results

We first present a summary of the overall findings identified to sustain the ongoing use of the Pain P/P as they relate to the three study objectives. Details of department and unit level findings were mapped to the DSF constructs (e.g., innovation, context or practice setting, broader system) and organized chronologically using the three time periods; implementation use phase (0–2 yrs), sustained use phase (>2–10 yrs), and 10 years post implementation (2017) (see Table 3). For each timeframe, we outline the study objective(s) the findings address, briefly describe the characteristics of the data sources, followed by the determinants and related KTIs reported by department and or unit level participants.

**Table 3:** Integrated case study findings for sustainability of pain BPG in acute care context.

**DSF themes/** **constructs**	**Integrated determinants (factors)** ***N*** **= 32**	***N*** = **32 unique determinants**			***N*** = **29 unique KTIs**		
		**Department RNs**	**Department RNs**	**Unit RNs**	**Department RNs**	**Department RNs**	**Unit RNs**
		**Implementation factors** **(0–2 yrs)**	**Sustained factors** **(>2–10 yrs)**	**Sustained factors** **(at 10 yrs)**	**Implementation phase (0–2 yrs)**	**Sustained phase** **(>2–10 yrs)**	**Sustained phase (at 10 yrs)**
		***n* = 3**	***n* = 12**	***n* = 31**	**KTIs (*n* = 12)**	**KTIs (*n* = 21)**	**KTIs (*n* = 9)**
		**3 ongoing determinants**			**8 ongoing KTIs**		
			**+10 unique factors**	**+19 unique factors**	**+ 4 unique implementation KTIs**	**+ 14 unique sustained KTIs**	**+ 3 unique sustained KTIs**
**Innovation** (defined as: new process/change/	^*^Relevance/consistent with competitive strategy (to addresses need/problem)	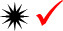		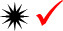			
product/practice or program, innovation, intervention)	Adaptability of innovation				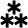 Embedding of Pain P/P into existing unit processes	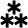 Embed ongoing refinements into existing routine practices/processes and Pain P/P	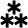 Routinize recommendations into nursing forms and practices/processes: embed prompts
							Digitalized Pain P/P and forms into new eHealth record
					Pain P/P established Interdisciplinary for all disciplines		
	Benefits to patient, staff, organization (cost effective, efficiency and quality of care)			✓1			
	Barrier identification				Use frameworks to guide implementation and Id barriers		
**Practice setting** (defined as inner context)	Human resources—recruitment, processes, succession and leave planning (staffing/compliment)		✓		Secure internal financial commitment—time and Human resources to participate on cttees and to implement KTIs		
	Student turnover (medical)		✓				
	Individual commitment to innovation			✓2			
	Individual competency (skill knowledge, absorptive capacity) to perform innovation and time management to use innovation			✓3			
	Expert consultants /resources			✓5			
	Internal cohesion between individual and commitment within the organization /stakeholder engagement leads to increased performance [senior nurse mentors /influencers vs. Clinical Care Leaders]			✓6			Mentorship used by senior nurses to support Pain P/P use:
	Stakeholder Commitment to innovation			**✓** **4**	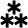 Joint collaboration of human resources from all levels of nursing plus other disciplines to develop departmental implementation plan		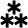 Engages IP stakeholder involvement: all professions to follow policy participate on cttees
	Stakeholder beliefs, attitude, perceptions, emotions, expectations toward innovation and user motivation/resistance		✓	✓			
	Population characteristic/needs/acuity level			✓13			
	Users awareness / familiarity with innovation			✓14			
**Practice setting** (defined as inner context)	Leadership commitment (department level)	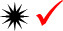	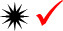		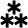 Formalize BPG Coordinator role	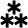 Comparing survey results among units created a sense of competition among leaders and users to improve	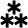 **Leadership strategies** - Clinical Coordinator- department level: (support for big issues during shifts) - Clinical Care Leaders—unit level (get involved in unit level issues to support ongoing improvements) - Unit Managers—unit level (get involved in unit wide issues, help with remedial action plans to reinforce target behaviors, review incidents, encourages education training)
	Management approach and engagement (commitment unit level)		**✓**	✓			
	Senior Leadership involvement and actions		**✓**				
Practice setting (defined as inner context)	Infrastructure support- Policies and Procedures based on Innovation (i.e., cttees, key people in nsg department– i.e., educators, champions, NPP reps)			✓7			
	Infrastructure support for innovation in job description with mechanism for recognizing achievement					Performance evaluation indicators for monitoring rt innovation= leaders, managers, and staff	
	Infrastructure support-equipment and supplies for innovation (and resources = pamphlets)			✓15			
	Physical layout/structure of wards			✓16			
	Competing corporate priorities		**✓**				
	Cultural—Beliefs, values and perceptions to innov			✓10			
	Cultural—Climate (doing research)			✓11			
	Cultural—innovation integrated into Norms (documents, protocols, manuals)			✓12		Unit leaders lead department and unit level patient centered initiatives for pain care based on unit routine practices -with adoption of EBP care	
	Team culture embraces innovation			**✓9**	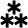 Obtaining buy-in and Formalize nurse leaders' involvement on Steering Cttee	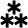 Corporate level Internal cttees' support ongoing review of clinical tactics support sustained use i.e., Patient Experience Steering cttee and Accreditation workgroup	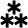 Fostering an IP and EBP culture among IP team to support Pain P/P use:
	Political internal stakeholder coalition, power, influence					Department determine EBP priorities	
	Financial performance budgeting and measurement				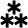 **Secure external funds** (a) RNAO PBSO—secure operating funds for initial training and resource s to build capacity (b) secure capital external financial support—for point of care surveying system	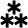 Development of an electronic monitoring system to measure nursing sensitive indicators provide monitoring of BPG adherence	
**Practice setting** (defined as inner context)	Workload /staffing patterns			✓17			
Practice setting (defined as inner context)	Education and training processes				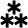 Pain Council established—Interdisciplinary taskforce leads initial policy development, education strategies and future policy revision	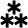 NPP reps develop formal and informal education initiatives at department and unit level in 2014 initially performed by the Pain Council.	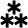 Ongoing education to support Pain P/P use by NPP and educators: - education days, - mandatory online modules - updates, refreshers, seminars
					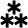 Educating Champions—to be clinical experts on units, with APNs	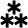 Trains 170 Unit level expertise to support use of Pain P/P s = Champions, educators, APNs, work across units as clinical resource	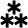 Ongoing Training to support Pain P/P use by NPP and educators: - general hospital orientation, - 1 on 1 training, in-services, solve recurrent problems
						Ongoing pain care education support at department and unit levels becomes tailored over time ie 1 on 1, case studies	
						Mandatory eLearn training system	
						Unit specific training of staff provided based on audit remedial action plans to improve on related BPG survey indicators	
						Develop unit specific additional resources/tools over time	
	Processual—planning, method, and timing of embedding innovation			✓18	Use multi-modal approach to disseminate		
	Processual—project structure and system to monitor/manage innovation					Spread EBP to additional areas	
					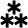 Established Pain BPG taskforce/workgroup in NPP department—enduring central reporting and monitoring structure for ongoing implementation and evaluation	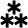 NPP and Unit Leaders facilitate/lead remedial action plan for underperforming units	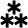 Monitoring and evaluation: Department level—ongoing training to do survey Unit level—audit and feedback provided (timely sharing of audit data, focuses biannual audit questions on target behaviors) Unit level—Patient satisfaction survey results shared reviews incidents and develop strategies to prevent them in staff mtgs
	Organization—communication capacity for monitoring (exchange and feedback)			**✓** **8**		Ongoing biannual training of staff to conduct prevalence survey	
						NPP Establishes regular performance monitoring: includes results from biannual prevalence audit and internal incident reporting	
						Timely exchange of prevalence survey results led to course correcting changes	
	**Formal communicating/reporting systems for client info btwn practitioners (documented)			19			Establishing effective communications between providers, reporting practices—bedside exchange, whiteboards, clipboards
Broader system (defined as: external condition, context, system, or environment)	External conditions, compatibility for innovation (consumer demand)		✓				
	External pressure/demand (e.g., professional/regulatory bodies, Ministry, funding bodies)	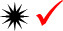	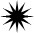	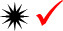		New evidence released—Integrating into BPG and ongoing education	
	Connection to broader external context (regional, national, international links)		✓			Staff participation on a regional network—provide access to new research and related outcomes for pain management	
	External support for innovation from stakeholders (recognition)		✓			Benchmarking to external sources best practices	
	**Goal alignment with external agencies (e.g., education institutes)		✓				

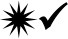
Determinants (factors) common across subcases over three timeframes.

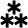
KTI common across subcases over three timeframes.

* represent common determinants across all 3 timeframes; 3 stars in a triangle shape represent common KTIs across all 3 timeframes; Green highlights represent common findings across timeframes; Blue highlights are used to separate 3 constructs (i.e. innovation, practice setting, broader system).

### Summary of overall findings

We identified a total of 32 unique determinants (*N* = 32) and 29 unique KTIs (*N* = 29) that influenced Pain P/P use over time (2007–2017), providing answers to study objective 1 (e.g., determinants influencing Pain P/P use) and 3 (e.g., KTIs influencing Pain P/P use), respectively. Notably, department and unit level nurses identified 3 determinants that continuously influenced Pain P/P use over all three time periods. This is a novel finding related to study objective 1. Department nurses separately identified 10 determinants that influenced Pain P/P use across all inpatient units during the sustained use phase (>2–10 yrs.). Two of these 10 determinants, along with an additional 19, were identified by unit nurses at the 10-year mark. Details related to determinants for all time periods, including supportive participant responses, and document evidence are available in Supplementary material 4.

Additionally, department and unit nurses described eight out of 29 KTIs that continuously promoted Pain P/P use over all three time periods. This is a novel finding related to study objective 3. An additional 4 KTIs were identified unique to the implementation use period (0–2 yrs.), 14 KTIs more unique to the sustained use period (>2–10 yrs.), and 3 KTIs unique only to the 10-year timeframe. Details related to KTIs, including supportive participant responses, and document evidence are available in Supplementary material 5.

At 10-years, audit results provided evidence that partially addressed study objective 2 (e.g., verifying unit nurses Pain P/P use 10 years post implementation), which were further explained by subcase nurses during interviews. Overall audit results revealed subcase nurses maintained high adherence rates for three out of five selected recommendations: namely R1-*assessing pain on admission to the unit*; R2- *once per shift and ongoing hourly assessments*, and R4-*establishing interventions to manage pain 10* years post initial implementation of the Pain P/P. Subcase nurses confirmed adherence to these recommendations was facilitated by innovation and context related KTIs. Furthermore, subcase nurses identified context related KTIs attributed to the low to moderate adherence rates evident by audit results for the remaining 2 selected recommendations: namely R3 –* establishing pain goals*; R7-*providing patient education related to pain management*.

### Implementation use phase (0–2 yrs)

#### Data sources

We interviewed three female department level Registered Nurses, who were part of the initial implementation team. Participants were involved in promoting the use of the Pain P/P over time (i.e., 2005–2017) while holding department-wide leadership positions, working across more than one nursing unit. Overall, there was consistency in their responses related to the determinants (i.e., objective 1) and the related KTIs (i.e., objective 3) influencing use of the Pain P/P recommendations during the implementation phase (0–2 yrs).

Documents collected provided a historical and organizational-wide perspective of the efforts used to sustain the Pain P/P's use across ~60 inpatient and outpatient units over time (2007–2017; see Supplementary material 3). Specifically, documents provided evidence that efforts were focused on policy and procedure development, training champions, assembling department infrastructure supports (e.g., Pain Council, interprofessional committees), followed by the use a multi-modal implementation approach led by NPP representatives and unit level champions.

#### Determinants

Department nurses identified the following 3 implementation determinants (1 innovation, 1 context, and 1 broader system) that influenced the hospital's decision to establish the Pain P/P as a “corporate-wide priority” in 2007:

(1) The *need for guideline* (innovation) to improve/standardize pain care based on patient satisfaction reports.
(2) Nursing *leaders' commitment* (context) to EBP use influenced Pain P/P use across all units.
(3) An *external demand* (broader system) by RNAO's call for proposals to establish a BPSO provided guideline recommendations, plus start-up funding to support efforts.

#### KTIs

Departmental nurses identified a total of 12 KTIs used across all inpatient units during the first 2 years. The following four KTIs (i–iv) were unique to the implementation phase (0–2 yrs.):

(i) *establishing an interdisciplinary policy* that applied to all disciplines including nursing;
(ii) *using knowledge translation models* they were familiar with, such as the Ottawa Model for Research Use (OMRU) (53) to guide guideline implementation, and the Knowledge to Action (KTA) framework (54) to assess potential barriers;
(iii) *allocating staff resources and time* to participate on BPG-IP committees and implement KTI initiatives across all units; and
(iv) using a *multi-modal dissemination approach* to initially train unit nurses which focused on providing education, development of assessment and documentation tools, and monitoring adherence.

The following eight KTIs (v to xii) promoted continuous use over all three time periods revealing how efforts evolved over time to address determinants influenced by changing underlying conditions:

(v) *obtaining buy-in from senior administration and formalizing their involvement* on a steering committee over the 10 years;
(vi) *getting joint collaboration from all levels of nursing* (Executive to point of care) and the engagement of other interprofessional stakeholders (i.e., Pharmacists, Therapists, and Medical Residents) in the development of ongoing implementation plans for all to follow influenced sustainment;
(vii) *establishing an interdisciplinary education and training structure*-a Pain Council/Taskforce that facilitated initial policy development, educational strategies and future direction for policy revisions. By 2014, these formal and informal departmental level education initiatives were assumed by NPP representatives and Champions. Over time, efforts by unit level Educators became more targeted to address unit level BPG adherence and related training needs;
(viii) *formalizing BPG-IP Coordinator role and related taskforces/workgroups* for each BPG within the NPP department;
(ix) *establishing a central reporting and monitoring structure* within the NPP department facilitated timely feedback of prevalence survey results to units and promoted formal reporting of unit level remedial plans designed to address low adherence rates. This monitoring structure reportedly “promoted ongoing use and evaluation” (P1);
(x) *embedding Pain P/P recommendations* using prompts and ongoing refinements *into already established documentation and quality care infrastructures* for hospital-wide implementation such as “general orientation” (P2), “mandatory eLearn modules” (P3), and “policy revisions” (P1) promoted high adherence rates during the first 2 years and over time;
(xi) *securing external financial support* from the RNAO facilitated training and access to a combination of external strategies to build capacity at the individual and department level (55). Securing external capital support of $30,000.00 from Canadian Nurse Foundation funded the development of an electronic point of care prevalence survey monitoring and evaluation system currently in use (56);
(xii) initially *educating* 60 practice *champions* to provide clinical expertise on pain care at both the department and unit levels. By 2017, 170 champions were trained.

### Sustained use phase (>2–10 yrs)

#### Data source

There was consistency in the responses of department nurses related to the determinants (i.e., objective 1) and the related KTIs (i.e., objective 3) influencing use of the Pain P/P recommendations during the sustained use phase (>2–10 yrs.) as well. Additionally, we interviewed 16 unit nurse participants (P), eight per subcase (e.g., Case 1: P1 to 8, Case 2: P1 to 8), seven female and one male per unit. Each unit had their own Manager, separate Educator and a mix of novice and senior unit nurses. Previous internal restructuring of the Medicine Care department resulted in both units being comprised of three inpatient wards, not all on the same floor, having approximately the same number of beds (e.g., 80 beds). Unit participants were Registered Nurses, the majority degree prepared (*n* = 13), between age 26 and 30 years of age (*n* = 9). Two participants in Subcase 1 and one participant in Subcase 2 were over 41 years of age. Subcase 1 nurses reported the average time working in the profession in their current job on their unit for 8 years, and Subcase 2 nurses reported the same for 9 years. No significant difference was noted between subcase nurses with respect to age (*p* = 0.599) or time in their current position (*p* = 0.823; see Table 4).

**Table 4:** Characteristics of unit level subcase participants.

**Key “subcase” participants**	**Case 1**	**Case 2**
Total participates (nurses)	*N* = 8	*N* = 8
**Current job title**		
Registered nurse	8	8
Female	7	7
Male	1	1
**Age distribution**		
26–30 yrs.	4	5
31–40 yrs.	2	2
41–50 yrs.	1	0
>50 yrs.	1	1
**Highest level of education**		
Diploma	2	1
Degree (bachelor degree in nursing)	6	7
**Time in the profession distribution**		
2.5–5 yrs.	3	4
6–10 yrs.	3	2
11–15 yrs.	1	1
>20 yrs.	1	1
Average time in the current job	8 yrs.	9 yrs.
	**Documents (see** **Supplementary material 3****)**	***N*** = **29**
	Reports	*N* = 7
	Internal documents	*N* = 20
	External documents	*N* = 2

To determine if differences existed between Subcase 1 and Subcase 2 groups, a Mann-Whitney U-test was conducted. Results indicated that there was no significant difference with respect to age across groups p-value = 0.599 or time in position across groups p-value = 0.823) (given p > 0.05).

Documents provided evidence that in 2010 and beyond, sustained use phase efforts focused on securing funds to purchase software, develop a point of care prevalence survey tool to evaluate use of BPG recommendations, and increasing unit nurses' adherence rates to selected BPG recommendations. Notably, between 2010 and 2015 the prevalence measures used to audit Pain P/P recommendations varied, targeting recommendations for short periods of time (e.g., 0–7 consecutive data points; see Supplementary material 3).

#### Determinants

We identified 10 determinants (6 context, 4 broader system) that influenced Pain P/P use across all inpatient units over time. Together, department and subcase nurses jointly identified the following six context determinants:

(1) “*Nurses' positive attitude* toward pain management and their commitment to quality… filtered throughout the hospital” (P1, P3) facilitating ongoing Pain P/P use.
(2) *Senior leadership's commitment* (i.e., Board of Directors) to leading a multi-disciplinary Quality Framework and working together on EBPs, influenced ongoing use.
(3) Together *department* (i.e., Chief Nursing Officer (CNO) and NPP representatives) and *unit level leaderships' commitment* (i.e., Educators, champions) supported ongoing use.
(4) Other *corporate priorities*, such as infection control rates, were identified as a barrier, temporarily refocusing attention from guideline adherence initiatives, competing with unit BPG priorities.
(5) A “*bimodal staffing complement* of novice and senior nurses on most inpatient units presented different ongoing education needs related to Pain P/P use” (P1).
(6) The constant *turnover of students* (e.g., medical, nursing) common in teaching hospitals, posed difficulties maintaining consistent practices between rotations.

The following 4 broader system determinants (7–10) were identified solely by department nurses:

(7) The local *university's goal* to use EBPs during medical and nursing student practicums *aligned with the hospital's goal*.
(8) *Increasing health consumer (patient) demand* for information on pain care management influenced nurses' active participation on internal committees.
(9) The RNAO's *formal recognition* of the electronic prevalence survey system encouraged ongoing accountability for BPGs.
(10) During the past decade the *increased focus internationally/nationally on Pain Care* broadened the knowledge base for nurses to draw upon.

#### KTIs

Department nurses uniquely identified 14 KTIs (11 context, 3 broader system,) used to sustain Pain P/P use across all units over time.

The 11 context KTIs included:

(i) By 2012, *units determined priorities for EBPs* based on inpatient needs and prevalence audit results;
(ii) Managers and Clinical Leaders lead the *integration of department and unit level patient centered EBPs into unit routine practices*, the latter varying between units;
(iii) In 2013, providing support for the development of *additional pain assessment tools* (e.g., Patient Information Booklets, verbal bedside shift reports, in room care boards with pain scales to communicate patient pain scores and goals, post-surgery pain management pamphlet) facilitated ongoing use of the Pain P/P on all units;
(iv) *sharing (e.g., spreading)* of pain practices/procedures and *tools* with outpatient departments;
(v) expanding efforts to provide *ongoing pain education* related to pain care and policy updates over time at the department and unit level (e.g., facilitating unit-wide case debriefings to resolve complex situations to offering 1 on 1 pain management training);
(vi) By 2013, the development and implementation of *mandatory pain care eLearn training modules* promoted ongoing Pain P/P use among new hires;
(vii) In 2014, Clinical Directors included “a *BPG-related performance criteria in evaluations* of their Clinical Nurse Leaders, who included the same in staff performance reviews” (P1). This specific KTI reportedly spurred the following four exchange and feedback KTIs (i.e., viii–xi);
(viii) providing *biannual prevalence training of staff to conduct the survey* “encouraged ongoing accountability internally for EBP process activities and results while building capacity” (P2);
(ix) NPP representatives began to provide regular performance results to units. The comparing of survey results among units *created a sense of competition* among unit leaders and staff to improve;
(x) timely exchange of results led to three incremental “*course-correcting changes*” (33):

- Measurements (e.g., survey questions) became more focused and sophisticated to target selected BPG behaviors. For example, leaders set increasingly specific benchmarks that were incrementally obtainable and modified survey questions to reflect benchmarks.
- Unit champions and Educators reportingly “designed KTIs to address targeted BPG behaviors evaluated” (P3).
- Survey methods expanded over time. For example, increasing numbers of nurses and interprofessional staff were trained to collect data on units not their own. This “increased awareness of BPGs and expanded accountability for patient safety performance among point of care practitioners” (P3); and

(xi) *under performing “unit teams”* and Clinical Nurse Leaders began to report back to NPP representatives on how they planned to respond to survey results” (P3) by *providing formal remedial action plans*. These monitoring and evaluative efforts “served to build nurses' problem-solving capacity and support continued Pain P/P use” (P1).

The 3 broader system KTIs (xii–xiv) included:

(xii) facilitating *staff participation on a regional network* to access new pain research;
(xiii) supporting the *integration of new evidence* (i.e., medication/treatment releases) into the Pain P/P; and
(xiv) *learning from and benchmarking to external sources* on best practices for pain care.

### Ten-years post implementation (2017)

To address study objective 2: verifying unit nurses Pain P/P use post implementation, a chart audit was conducted. To address study objectives 1 (i.e., identifying determinants) and 3 (i.e., identifying KTIs) we interviewed subcase nurses. Similarities and differences identified among the two subcases are presented.

#### Data sources

Chart audit results revealed similar patient profiles were admitted to each subcase unit (see Table 5). Female patients represented 55 and 58% of admissions for Subcase 1 and 2, respectively. The average patient age was 72. Patients were admitted from the emergency department, except two for subcase 1, and six for subcase 2. The primary admission diagnosis was decline/failure to cope/generalized weakness, referred to as non-specific, followed by a respiratory diagnosis. All other admission diagnoses included system related illness (e.g., cardiac). Length of stay was 9 days for Subcase 1, and 11 days for Subcase 2 patients.

**Table 5:** Patient profiles included in chart audit by subcase.

**Subcases**		**‘Case 1' vs Subcase 1**	**‘Case 2' vs subcase 2**
Dates		Aug -Oct 2016, Jan-Mar 2017, Jul-Oct 2017	Aug -Oct 2016, Jan-Mar 2017, Jul-Oct 2017
Male admissions to unit		45	42	
Female admissions to unit		55	58	
Patient average age		72 yrs. old	72 yrs. old	
1st	Other	44	38	^*^Non-specific
2nd	Respiratory	23	21	Respiratory
3rd	NYD	13		
3rd			12	Gastrointestinal
3rd			12	Neurological
4th	Cardiac	10	6	Cardiac
5th	Musculoskeletal	5	4	Musculoskeletal
5th	Gastrointestinal	5		
5th			4	NYD
6th			3	Cancer
**ALOS**		**8.6 days**	**11.4 days**	
Emergency to medicine		98	94	
Directly to medicine		0	3	
ICU/urgent care to medicine		2	2	
Endoscopy to medicine		0	1	

* Non-specific—decline/failure to cope/Altered LOC/ confusion/general weakness.

Chart audit results (see Table 6) provided evidence for study objective 2 indicating subcase nurses maintained high adherence levels (>80% of the time) (48) to three of the five recommendations (R) 10 years post implementation: R1-*assessing pain on admission to the unit* (R1), R2- *once per shift and ongoing hourly assessments*, and R4-*establishing interventions to manage pain*. Low adherence rates (<50%) (48) existed across subcases for R7-*providing patient education related to pain management*. There was a significant difference in the adherence rate for R3-*to establishing Pain Goal*(s) for patients who had pain during their hospital stay (over five shifts); Subcases 1 (C1) had low adherence, and Subcase 2 (C2) had moderate adherence (between 50 and 80%) (48).

**Table 6:** Audit results for subcases' adherence rates to Pain P/P recommendations.

**Recommendation**		**Case 1 (C1)**	**Case 2 (C2)**	**Adherence rate**
R1	Pain assessment on admission to unit (shift 1) Range of pain scores = 0–10	98% (98/100) charts had initial assessment on unit admission history. 2/100 charts had missing data	99% (99/100) charts had initial assessment on unit admission history. • 1/100 charts had missing data	High adherence to R1
R2	Ongoing pain assessment (shifts 2–5)	98.5% (98.5/100) charts/four shifts had ongoing pain assessment for next four shifts • 1.5 /100 charts/shift had missing data • 98.75% (98.75/100) charts/five shifts had hourly round checks completed	98% (98/100) charts/four shifts had ongoing pain assessment for next four shifts • 2/100 charts/shift had missing data • 99.5% (99.5/100) charts/five shifts had hourly round checks completed	High adherence to R2 • Once per shift and hourly rounds • Hourly rounds—no documented pain scores
R3	Establishes Pain Goal for patients who had pain during stay (over five shifts)	R3-19/53 (36%) charts of patients who had pain score >0 had Pain Goal set during stay evidence in IPN and or progress notes. • 9/19 collaborated with pt on PG • 10/19 had pain scores ≥4	R3-32/55 (58%) charts of patients who had pain score >0 had Pain Goal set during stay evidence in IPN and or progress notes. • 17/32 collaborated with pt on PG • 22/32 had pain scores ≥4	R3 C1- Low adherence to setting of Pain Goal 1 on admission history • R3 C2- Moderate adherence to setting Pain Goal 2 during stay
R4	Establishment of interventions to manage pain for patients with pain	52/53(98%) charts of patients who had pain score >0 had evidence of prescribed interventions to manage pain • 35/53 charts only prescribed Pharm • 12/53 charts with combo of prescribed Pharm and Non-Pharm interventions • 3/53 charts prescribed Pharm+Methadone • 2/53 charts with prescribed Pharm prn • 1/53 no intervention	55/55 (100%) charts of patients who had pain score >0 had evidence of prescribed interventions to manage pain • 45/55 charts only prescribed Pharm • 9/55 charts with combo of prescribed Pharm and Non-Pharm interventions • 0/55 charts prescribed Pharm+Methadone • 1/55 charts with prescribed Pharm prn • 0/55 no intervention	High adherence to establishing pain mgmt interventions
R7	Patient or family education related to pain management for patients with pain	0/53 (0%) charts with Pt. Education Form • 0 /53 (0%) charts with evidence of pt education on pain mgmt provided in IPN	• 1/55 (2%) charts with Pt. Education Form (re: Atrovent and neb use)[] • 0 /55 (0%) charts with evidence of pt education on pain mgmt provided in IPN	• Low adherence • No use of Pt Education Form. • No documented evidence of “Pt education” provided on pain management plan in IPN.

High adherence rate (>80%), moderate adherence rate (50−80%), and low adherence rate (< 50%) of the time (48).

#### Determinants

We identified 19 additional determinants (1 innovation, 18 context) subcase (C1, C2) participants (P) stated influenced their Pain P/P use at the 10-year timeframe, addressing study objective 1 (e.g., determinants).

Subcases nurses identified 1 innovation determinant:

(1) Nurses described how Pain P/P use *benefited patients* stating, “we can make the most difference... noticing (assessing) if my patients are in pain and advocating for …prns …using the Pain P/P” (C2:P4).

Subcase nurses identified the following 18 context (2–19) determinants:

(2) Nurses reported their *commitment to the innovation* influenced their use of the Pain P/P, declaring “we are very supportive of the use of evidence-based practices, like the Pain P/P” (C1:P3).
(3) Nurses also indicated they felt *competent providing pain care* stating “we have the knowledge and skill to use the Pain P/P, as it [pain care] has been ingrained in us for a very long time, …since our training” (C1:P1).
(4) Nurses claimed “the *commitment of multiple stakeholders* such as managers” (C1:P5) …and “healthcare aides” (C2:P4) influenced their use of the Pain P/P.
(5) Having access to *collaborative expert consultants* (e.g., Acute Pain services, Palliative Care Services) to “deal with difficult pain care situations…when MDs can't control patients' pain” (C1:P1) supported Pain P/P use.
(6) The *internal cohesion between individuals* on the units (subcases) and their commitment to the Pain P/P differed among subcases, yet both promoted Pain P/P use. For example, Subcase 1 nurses claimed their clinical leaders (manager and educator) mainly influenced their use of the Pain P/P. Whereas, Subcase 2 nurses emphasized *senior nurse mentors* influenced their competency/skill performing pain care avowing “everything I have learned about pain control has come from senior nurses” (C2:P6).
(7) Nurses confirmed the *presence of infrastructure support* within the nursing department (i.e., committees/workgroups, educators, champions, NPP representatives) influenced use over time.
(8) The use of the following processual methods such as “hourly rounding, bedside shift reports” (C1: P6) and “in room care boards” (C2:P4) reportedly influenced *enhanced communications (exchange and feedback)* related to patients' pain status and management among nurses.
(9) The existence of a *team culture* on committees throughout the hospital that embraced new initiatives/approaches to pain management encouraged nurses' “openness to use alternative therapies” (C1:P5) and evidence-informed “new treatment modalities” (C2:P2).
(10) Subcase nurses indicated the clinical Managers were key to fostering *an EBP culture (beliefs, values, perceptions) toward pain care* on their units “supporting and encouraging them to attend pain education days, conferences related to new meds, techniques, and ways to control pain” (C2:P6).
(11) Five subcase 1 nurses claimed their Manager's focus on improving pain care was supported by *a climate for doing research* on the unit, encouraging “one nurse to do her Masters on non-verbal pain indicators …on the unit” (C1:P5).
(12) Subcase 2 nurses stated, they “work within a very close dynamic interprofessional team” (C2:P8) that *integrated pain care into unit norms*; such as “patient daily rounds” (C2:P2), which influenced their use of the Pain P/P.

Subcase nurses identified seven barriers to Pain P/P use at the 10-year mark:

(13) *Patient/family characteristics* influenced their use of the Pain P/P admitting “assessing pain is challenging when patients are afraid of taking pain medication” (C2:P1), and or “if families are scared to ask for medications” (C1:P8).
(14) A lack of user *familiarity/awareness of the Pain P/P* indicating, they “don't think many people refer to it [Pain P/P] beyond orientation” (C1:P5), nor were aware “it was an actual legit document” (C2:P6).
(15) Subcase 2 nurses indicated the *lack of available pain management resources* on the unit, such as “a formal clinical pathway for pain control” (C2:P4) or “pain standing orders” (C2:P3) as a barrier. Nurses further indicated there was a need for unit in-services on *specialized equipment*, like CADD pain pumps” (C2:P8) on the units.
(16) The recent internal unit restructuring was identified as a barrier. Specifically, the *physical structure/layout* was “more than one floor” (C2:P8), and “too large, containing more than 80 beds” (C1:P1).
(17) Nurses indicated increased *workload* or decreased *staffing ratios* was a barrier, explaining “assigning one nurse to six patients is too much to maintain and control pain levels” (C2:P6).
(18) The *utility of the new electronic patient information charting (EPIC) system* was identified as barrier. Nurses stated “it's [EPIC] so frustrating going back and forth from the bedside to the EPIC system to scan your patient, then go back to the med cart to get your meds” (C1:P7).
(19) Nurses stated use of the established Education Form: a *formal information reporting system between practitioners* “was an unrealistic charting expectation” (C1:P7) revealing “we do education all the time, but don't document it, even on that form” (C2:P2). Additionally, nurses claimed a lack of MD and nurse communication related to the pain care they provide existed, indicating “very rarely do physicians prompt nurses about patient pain” (C2:P2).

#### KTIs

Three unique KTIs (1 innovation, 2 context) were identified by subcases that facilitated Pain P/P use at the 10-year mark, addressing study objective 3.

The 1 innovation KTI included:

(i) *digitalizing of Pain P/P recommendation prompts* into the new EPIC system promoted use.

The 2 context KTIs (ii-iii) included:

(ii) *senior nurse mentorship* of novice nurses' Pain P/P use “especially in pain crisis situations” (C2:P2), and providing “tips on non-verbal pain assessment and management techniques at bedside” (C1:P4, C2:P2); and
(iii) *establishing communication practices between providers* to report on patients' pain status (e.g., verbal bedside shift reports, documentation on patient care boards, vital sign clipboards) facilitated Pain P/P use at the 10-year mark.

## Discussion

Our findings provide insight into the sustainability of a Pain BPG, from a nursing department and unit level, within an acute care context. We identified a total of 32 sustainability determinants that address study objective 1, and 29 sustainability-orientated KTIs that fostered innovation sustainment in an acute care context over 10 years addressing study objective 3, These findings not only provide a listing of sustainability determinants and related KTIs for those planning or implementing BPGs in clinical practice, but more importantly the pairing of the KTIs to the determinants; whether a facilitator or barrier, to promote sustained use over time adds to the current knowledge (see Table 3).

In addition to identifying sustainability determinants and related KTIs, several key observations related to study findings are presented. For example novel findings revealed three determinants had a continuous influence during the implementation and sustained use phases. Unique determinants identified by department and unit nurses not only reflected changing context influences over time but a perspective based on their respective roles and responsibilities to the innovation. Unit nurses demonstrated a range of high to low adherence to the five selected guideline recommendations at the 10-year mark, addressing study objective 2. Combined department-wide level KTI efforts designed to standardize nursing documentation and unit level processes/practices contributed to these rates. Another novel finding revealed eight KTIs that continuously influenced Pain P/P use in implementation and sustained use phases. Lastly, five key observations related to the KTIs that were paramount to resolving the fit between the innovation (Pain P/P) and the changing context, during both phases are presented.

### Three determinants having continuous influence over time

Our research provides insight into the relationship among three determinants across both phases important for sustainment: (i) a *need;* (ii) *external demand*, and (iii) *leadership commitment*. Although these determinants have been identified for sustainment of EBPs (5, 8, 14, 57–59), our study provides novel evidence of the potential impact of implementation determinants on sustainability of innovations in acute care context recently proposed in the literature (58, 60). The following discussion examines the influences underlying these three determinants over time and their impact on sustainment.

During the implementation phase (0–2 yrs.) a *need* was identified among department nurses to ensure a consistent approach to pain care across all inpatient units facilitating the development of an interdisciplinary Pain P/P (i.e., innovation) designed for all disciplines to follow. Whereas, during the sustained phase (>2–10 yrs.) *ongoing need* for the innovation by internal stakeholders (i.e., inpatients) at the clinical level (unit) contributed to sustained use. Department and unit nurses' ongoing perception of the innovation's need, its' safety and quality, and over time its' relevance to addressing a need (perceived benefit to patients) reportingly influenced ongoing use. This finding is congruent with the evidence in the literature (14, 16, 61). Similarly, expectations (*external demand*) from healthcare regulatory bodies on hospital leaders to embrace evidence-based care in the implementation phase, over time shifted to a requirement by the Ministry and accrediting bodies to report related quality and standards of care data. Brewster et al. (61) purports efforts such as these “transform innovations from a practice imposed on an organizational system, to habits that are reinforced by the system” (61). Thus, external pressure/demand eventually took on the role of holding the EBP in place, promoting sustainment of the Pain P/P over time and at the 10-year timeframe. Lastly, the combination of *leadership commitment* expanded over time to include both department and unit level leaders as the focus on Pain P/P use moved from a department level (implementation phase) to the clinical practice level (sustained phase). Leadership engagement at all levels is identified in previous studies as a key factor influencing sustainment (1, 3, 21, 24, 62).

Clearly, attention to these three determinants and how they influenced use of the Pain P/P during both phases, at multiple levels, was necessary for sustainment. This finding provides evidence that changing conditions (e.g., level of application) do impact not only the fit between the innovation and the context, but ongoing use over time corroborated by other researchers (6, 63, 64). The fact that the underlying condition influencing these determinants did evolve over time further supports the conceptualization of sustainability as an “*ongoing dynamic process*” (58). Thus, we recommend these determinants be considered early in the knowledge to action cycle when planning and in the development of sustainability action plans indicated by other researchers (1, 57, 60, 65, 66).

### Unique department and unit nurses' determinants

Together, department and unit level nurses identified 32 sustainability determinants that not only addressed study objective 1, but revealed insights not anticipated. Specifically, unique determinants identified by the department and unit nurses reflected a viewpoint based on their respective roles and responsibilities related to the innovation. For example, department nurses reported broader system (e.g., connections with external networks) and organizational-wide practice setting (e.g., internal competing priorities) determinants impacting sustainability over time. These determinants reflect an “outward focus” and insight into their roles and responsibilities across all units which positioned them “to act as conduits, linking outer and inner contextual influences” to ensure sustainment of the innovation over time in a changing context. This finding adds to the nurse leadership roles identified in a previous study wherein the mid-level management role is described as being critical to enacting a tie between the unit level leaders and point of care (24).

Conversely, determinants identified by unit nurses, focused mainly on the “innovation” and how it meets patient needs, and nurses' use of it within their daily practice, related structures and processes on the unit: the local context. Unit nurses identified that “patient/family perceived benefits of an innovation” influenced their use of BPGs. This finding aligns with a recent study wherein hospital-based nurses reported continued benefits as an essential innovation characteristic for sustainability of BPGs (15). Researchers further suggest provider collaboration as a key determinant influencing the implementation of BPGs in hospitals (67, 68). A novel finding in our study stems from the linkages/interactions between and attributes of unit level leaders, senior nurse mentors and interprofessional team members on the subcase units. The literature suggests dynamic elements of context, such as increasing complexity and acuity of inpatients, often requires interdependence among nursing colleagues and other interprofessional team practitioners to maintain BPGs (67). Unit nurses reinforced how nursing work is dependent on linkages within the network of care it is located in (e.g., between the persons and clinical processes on the unit) noted in a previous study (15) which impacted their sustained use of BPGs. Thus, despite differences in supervision (e.g., unit leaders) and organization culture/climate (mentors and IP team members) determinants, the linkages/interactions between and attributes of these key individuals are important for sustainability, which has not been previously reported, adding to current knowledge.

### Adherence to selected guideline recommendations

Findings related to study objective 2 revealed a range of high to low adherence rates to the selected five recommendations among subcase nurses 10 years post implementation. Specifically, unit nurses demonstrated high adherence to three recommendations: R1 (assessment on admission), R2 (assessment once per shift and hourly rounds), and R4 (establishment of interventions to manage pain). These findings further support evidence in the literature that standardized documentation practices (69), the integrations of recommendations into daily processes and practice routines (70), and ongoing audit and feedback related to guideline recommendations (70) promotes formal documentation of recommendations necessary to accurately measure sustainment. It is unclear if one or the combination of all efforts made a difference. Likely, over time all played a role.

Given our findings, we cannot say with certainty there is an evidence-practice gap for recommendations R3 (setting pain goals) and R7 (educating patients/families regarding their pain management plan) 10 years post implementation. Although we found a significant difference in adherence to R3 (i.e., moderate adherence) establishing pain goals on subcase 2 compared to subcase 1 (i.e., low adherence), findings revealed unit level practices (e.g., use of whiteboards and bedside shift reports) influenced nurses' lack of documentation in the clinical records. Similarly, for R7, although no formal documentation (i.e., on Patient Education Form) was evident indicating patients received pain education, nurses indicated they provided pain care education all the time. The accuracy of nursing documentation among acute care nurses has previously been studied (71, 72). These studies have reported low scores on (i) the accuracy of nursing intervention documentation (71, 72) and (ii) that nurses' documented EBP “assessments of patient status” more frequently than the “nursing interventions they were preforming” (72). Uncovering informal processes at point of care for recommendations exhibiting moderate to low adherence rates is necessary in order to develop effective KTIs to promote accurate documentation of nursing interventions to effectively measure sustainment.

### Eight sustainability KTIs used over 10 years

We identified a total of 29 sustainability-orientated KTIs that influenced the ongoing fit between the innovation (Pain P/P) and the changing context which addressed study objective 3. Another novel finding in this study is both department and unit nurses described eight KTIs that continuously promoted the use of the Pain P/P over 10 years. These eight KTIs provided insight into how the focus of the KTIs evolved over time with the change in level of application (e.g., across units/department vs. unit specific application). This novel finding is important to consider when designing KTIs to be used in ever-changing healthcare settings. Our findings demonstrate **s**ustainability requires continual efforts but if undertaken as an integrated part of improving overall institutional performance, can create a supportive climate for EBP sustainment. Given the continued impact of the eight KTIs over time we recommend they be considered early in the planning stage for those aiming to sustain BPGs in similar acute care settings.

### Key observations related to sustainability-orientated KTIs

Five key observations about KTIs that we perceive fostered changed behaviors and facilitated sustainment overtime in our study are: (i) two implementation KTIs had an enduring impact in both phases; (ii) the linking of KTIs to one recommendation at time (e.g., an incremental approach) promoted sustainment; (iii) use of a participatory approach that engaged leaders and unit nurses in the development of KTIs; (iv) development of an infrastructure to monitor adherence that engaged nurses promoted accountability for EB care and built capacity, and (v) creating an institutional system that held leadership accountable for EBP outcomes.

First, two implementation phase KTIs that had an enduring impact in both phases were: the *use of frameworks* and *securing external financial resources* for the BPG-IP. Using “framework-inspired method” (e.g., KTA and OMRU) (53) to “facilitate early identification of barriers” (65) and to tailor KTIs is a creative way to provide guidance on how to proceed, while promoting stakeholder engagement and interest in facilitating ongoing decision-making, to ensure sustainability of EBPs (65). This recommendation corroborates that of other researchers (14, 18, 58, 73–75). *Securing external financial resources* to develop an “electronic point of care prevalence monitoring system” that measured nursing sensitive indicators beyond implementation, was recognized externally as a key sustainability-orientated KTI. Securing funds to support innovation initiatives is congruent with existing sustainability frameworks (14, 16).

Second, findings revealed the adapting and refinement of EBPs to local context over time also requires continual efforts focused on designing KTIs that address changing contextual influences to promote ongoing use. Specifically, during the implementation phase, KTIs were focused on integrating recommendations into existing organization-wide documentation and orientation processes/practices. During the sustained phase, the focus and design of KTIs changed to address unit specific low adherence rates. This change likely stemmed from the realization they could not obtain high adherence to all BPG recommendations on all units at the same time. The added value or effectiveness of tailoring KTIs overtime to support the integration of the innovation into routine practices/processes (in context), previously identified as an implementation strategy to overcome barriers to change (76, 77), now adds to sustainability knowledge. These findings further reinforce that a balance is needed between maintaining ongoing organization KTIs and allowing units the latitude to link KTIs, designed specifically to address unit specific low adherence rates, to facilitate successful sustainment. This novel finding substantiates that innovation sustainability is broader than just maintaining the fidelity of the original EBP (Pain P/P) but instead one that exhibits ongoing continuous adjustments and refinements to optimize its utility within a changing context (6). Our findings also add credence to the conceptualization that sustainability of healthcare innovations in clinical practice is as an “*ongoing dynamic process*” (58).

The third, involves the use of a participatory approach that engaged point of care users in the development of KTIs to enhance adherence: a bottom-up participatory approach. This strategy effectively built on their successes related to guideline adherence rates while continuing to improve patient outcomes. These findings confirm the notion that to produce real world change over time there is a “need to consider staff and system domains as active components in the change process rather than imposing change” (4). This active participatory and incremental approach to develop strategies by unit level users (4, 78–80), led by clinical leaders (33, 79) contributed to sustainment in the changing acute care context.

A fourth observation involves the combining of two KTIs (e.g., monitoring and training) designed to promote accountability while building capacity for evidence-based care. In a recent review of sustainability approaches used to sustain innovations in healthcare “monitoring progress overtime” emerged as “a consistent construct across approaches regardless of the proposed innovation, settings or application types” (79). Efforts by the study site to establish a point of care *monitoring and feedback system*, that provided *regular reports* on nurses' *adherence rates* to BPG recommendations produced the necessary data critical to determine *unit level remedial action plans* (e.g., feedback mechanisms). These efforts reportedly contributed to sustainment and have been reported by others (79, 81). Additionally, the training of users to conduct the surveys and engage in feedback processes reportedly *enhanced capacity to monitor progress* overtime contributing to sustained use. These KTIs should be considered by those planning or in the process of creating a sustainability monitoring infrastructure system.

Fifth, the integrations of a *BPG-related performance criterion* into the performance evaluation system had a trickledown effect into the nurse manager and subsequent unit nurses' performance expectations and was critical to the process of change (e.g., adherence to guideline recommendations) and likelihood of sustained use over time. This KTI focused on obtaining shared accountability (e.g., getting buy-in) to deliver the innovation [Pain P/P] in support of the departments' vision for EB care. This finding is congruent with a study wherein point of care nursing leaders promoted shared accountability by reinforcing the expectation of BPG as the practice standard on their units (15, 24). Consistent reinforcement and evaluation of guideline standards by leaders with teams of nurses was a key KTI consideration for sustained use of BPGs in our study.

### Strengths and limitations

To our knowledge this is the first study to provide theory-informed, in-depth, contextualized evidence about the determinants and related KTIs used over a 10-year timeframe to sustain the use of a nursing guideline in acute care. Novel insights related to the relationship between determinants and KTIs and their level of application (department and unit levels) over time were revealed. Detail and in-depth descriptions needed to determine the extent or transferability of our findings to similar settings is provided. We used multiple forms of data, conducted debriefings with the research team, and substantiated findings with knowledge users to enhance credibility. Adhering to the study protocol, documenting decision points, maintaining organized paper and electronic databases, and maintaining a master list of definitions, questions, and codes enhanced dependability. Referencing multiple data sources, remaining close to participant verbatim transcripts, and demonstrating data congruency between two or more participants ensured confirmability.

Limitations include the possibility of non-response and recall bias among department level nurses given the retrospective nature of interview process. Although the interviews occurred at the 10-year mark, participants remembered details from start to present day, given they remain currently engaged in ongoing efforts to support sustainment. Other potential biases include sampling, participant social response bias, and potential researcher bias. Sample selection was limited given the capacity of the primary researcher who collected all data. Subcase selection was based on maximum variation criteria providing potentially contrasting patterns of findings established by internal representatives and voluntary participation. Furthermore, including additional subcases (units) in future sampling may provide further insights and or confirm findings. Social response bias may have occurred if participants' responses to the interview questions indicated what they thought would be acceptable rather than their perspective. Steps taken to decrease social response biases included triangulating data sources and validating themes within the qualitative analysis. To reduce researchers' bias, we used multiple data sources and substantiated findings with knowledge users. Finally, the examination of one BPG, within one multi-site healthcare organization, from solely a nursing perspective, is a limitation. Given the Pain P/P is an interdisciplinary policy, perceptions from medical and allied health professionals, other than department and unit level nurses were not included and may have.

## Conclusion

Sustainability of EBPs in acute care has been recognized as a challenge. Together, determinants and KTIs influence the way in which healthcare innovations are sustained over time. It is important to understand the influences underlying the determinants in real world settings and how the focus of the KTIs must evolve over time with the integration of an innovation at different levels of application (e.g., department vs. unit level). KTIs that fostered behavior changes to sustain a BPG were paramount to resolving the fit between the innovation and the changing context over time. Given healthcare innovation sustainability is a “process” or “ongoing stage,” it is noticeable from these findings what really matters is how and what the organization does to sustain the innovation at all levels over time within ever-changing acute care contexts. Future inquiry needs to focus on examining KTIs that promote documentation of nursing interventions related to recommendations (e.g., R4-setting pain goals, R7-providing patient/family pain management education) which revealed low to moderate adherence rates. To further our understanding of sustainability, qualitative methodologies should be used to uncover unit level determinants and KTIs underlying nurses' adherence to guideline recommendations across a range of healthcare settings with the intention of adding to the existing sustainability knowledge base.

## Data availability statement

The original contributions presented in the study are included in the article/Supplementary material, further inquiries can be directed to the corresponding author.

## Ethics statement

The studies involving human participants were reviewed and approved by Research Ethics Boards for the Ottawa Health Science Network (OHSN-REB) and the University of Ottawa. The patients/participants provided their written informed consent to participate in this study.

## Author contributions

LNP, IG, BD, CB, and JS conceived the study design. LNP was responsible for the data collection, synthesis, conducted the quantitative data analysis, and produced all tables, figures, and Supplementary materials. LNP and JF-P conducted analysis of qualitative data. JS, IG, and CB provided input into the analysis and interpretation. The initial draft of the manuscript was prepared by LNP as part of dissertation research and then circulated among all coauthors for comments and revision. All authors read and approved the final manuscript.

## Funding

IG is a recipient of a CIHR Foundation Grant, FDN#143237.

## Conflict of interest

The authors declare that the research was conducted in the absence of any commercial or financial relationships that could be construed as a potential conflict of interest.

## Publisher's note

All claims expressed in this article are solely those of the authors and do not necessarily represent those of their affiliated organizations, or those of the publisher, the editors and the reviewers. Any product that may be evaluated in this article, or claim that may be made by its manufacturer, is not guaranteed or endorsed by the publisher.

## Abbreviations

APN: Advance Practice Nurse
BPG: Best Practice Guideline
APS: Acute Pain Service
BPG-IP: Best Practice Guideline-Implementation Program
BPSO: Best Practice Spotlight Organization
C1:P#, Subcase-1: participant /informant code
C2:P#, Subcase-2: participant /informant code
CAN: Canadian Association of Nurses
CNO: College of Nurses of Ontario
DSF: Dynamic Sustainability Framework
EBP: Evidence based practices
ED#: External document code numbered 1 to 2
ID#: Internal document code numbered 1 to 20
KTIs: Knowledge translation interventions
NPP: Nursing Professional Practice
P#: participant informant /code
Pain P/P: Pain policy/protocol
Rt#: Report code numbered 1 to 7
RNAO: Registered Nurses&#8217 Association of Ontario.
