# Supplementary material for: Sustaining a nursing best practice guideline in an acute care setting over 10 years: A mixed methods case study
Source: Front Health Serv. 2022 Aug 30;2:940936. doi: 10.3389/frhs.2022.940936 (PMC10012662; doi:10.3389/frhs.2022.940936)
Supplement: Supplementary file 1 [file Table_1.docx]

**Supplementary material 1.** Mixed Methods Article Reporting Standards (MMARS)

| **Standard** | **Line Number** |
| --- | --- |
| Title  Authors information | Line 57-65  Line 66-73 |
| **Abstract** | Line 74-171 |
| **Introduction**  Background  Description of Research Problem /Questions | Line 121-197  Line 198-219 |
| Study aims/research Goals | Line 220-231 |
| **Method**  Research Design  Reflexivity statement  Setting | Line 243-265  Line 231-239  Line 266-274 |
| ***Participant sampling or selection***  Quantitative (subunits for audit)  Qualitative  Department level,  Unit level | Line 434-440  Line 474-510  Line 518-525 |
| ***Participant recruitment***  Quantitative (Audit)  Qualitative  Department level,  Unit level | Line 422-431  Line 510-514  Line 525-533 |
| **Data collection/Identification Procedures**  Quantitative (Documents and Audit)  Qualitative  Department level  Unit level | Line 440-448  Line 317-418  Line 515-533 |
| **Recording and Transforming the Data**  Quantitative (Document, Audit)  Qualitative  Department level,  Unit level | Line 423-431, 448-463  Line 467-514  Line 525-533 |
| **Data Analysis**  Quantitative  Qualitative | Line 538-572  Line 575-628 |
| **Validity, Reliability and Methodological Integrity**  Quantitative  Qualitative  Ethics | Line 561-572, Figure 1  Line 576-628, Table 2  Line 631-646 |
| **Findings/Results**  Summary of Overall Findings  Quantitative Results (Documents, Audit)  Qualitative Findings  Department  Unit | Line 650-707  Line 722-731, 2011-2055, 2197-2209.  Line 713-721, 735-1985, 1991-1995, 2060-2173.  Line 1995-2010, 2060-2084, 2176-2181, 2214-2497. |
| **Discussion**  Contributions  Determinant- Interpretations and implications  Adherence to Recommendations  KTIs- Interpretation and implications  Future research | Line 2500-2531  Line 2534-2638  Line 2642-2682  Line -2686-2809  Line 2875-2884 |
| **Strengths and Limitation**  Strengths  Limitations | Line 2812-2829  Line 2830-2856 |
| **Conclusions** | Line 2860-2884 |
