# Supplementary material for: Sustaining a nursing best practice guideline in an acute care setting over 10 years: A mixed methods case study
Source: Front Health Serv. 2022 Aug 30;2:940936. doi: 10.3389/frhs.2022.940936 (PMC10012662; doi:10.3389/frhs.2022.940936)
Supplement: Supplementary file 2 [file Table_2.docx]

**Supplementary material 2.** Dynamic Sustainability Framework (1) and its application to the case study

T1, T2, T3 = measures Pain P/P use at Department Level

T4, T6, T7 = measures Pain P/P use at Department and Unit level

T5 =measures Pain P/P use at Unit Level

| **DSF TENET/**  **PROPOSITION**  **(Chambers et al 2013)** | **DSF Label** | **Short User friendly explanation** | **Specific Example** | **Application to this Case Study**  **(my interpretation)** | **Draft Qualitative Interview Questions** |
| --- | --- | --- | --- | --- | --- |
| 1. Optimizing of HC Int (Pain P/P) is context specific and shd not be optimized prior to Implementation (0-2 yrs.) (Imp) and sustainability (Sust) (> 2ys) phase onset.   FOCUS is on 0-2 yrs | **Corporate Level optimization 0-2 yrs.**  Flexibility & Optimization of Pain P/P... before embedded into routine practices = Imp phase  Ongoing continuous adjustments and refinements is the ultimate aim to optimize Pain P/P’s utility within a chging context vs maintaining fidelity of original protocol (QA) approach | Refers to how the Pain P/P was optimized for use in site (Corp level), then for dept. level & unit specific practices btwn 0-2 yrs. | Examine how Pain P/P was developed from RNAO BPG in 2005-7, then implemented into units 0-2 yrs. activities/efforts | Phase I = conduct corporate level document review... and interview corporate level nurses involved in the Implementation of the Pain P/P  ...look for answers to the initial development process and refinements between 0 to 2 yrs. of implementation. | **Department Level refinements (draft Qs)**   - 1. How did the hospital or Steering cttee decide on the RNAO BPG?   2. How was the Pain P/P developed?   3. How did they determine the best way to integrate/embed the use of the Pain P/P throughout the hospital?   4. How long did it take to finalize implementation of the Pain P/P throughout the hospital?   5. How was the Pain P/P refined or adjusted to be embedded into your routine practices within your hospital btwn 0-2 yrs? |
| **DSF TENET/**  **PROPOSITION** | **DSF Label** | **Short User friendly explanation** | **Specific Example** | **Application to this Case Study**  **Phase of Study**  **Data Sources** | **Draft Questions** |
| 1. Continual improvements of HC Intervention (i.e., Pain P/P) will boost sustainment   Focus is on >2yrs | **Corporate Level evolvement of Intervention (Int) >2yrs**  Pain P/P evolvement over time is needed to adapt it for sustained use in hospital  Intervention adaption is expected | Refers to how the policy has evolved or adapted overtime ...based on further local optimizations or refinements  Emphasizes the importance of streamlining HC Int by removing cpnts that are not central to improving outcomes or to adapt cpnts to better fit with the context to improve outcomes | Pain P/P changes between 2007 to 2013...  ...and what is going now to refine it. | Phase I = confirm usage with ...  Document review,  Phase II -  Interviews with Department nurse –part of original Steering cttee | **Department Level adaption of Pain P/P**   - 1. What adjustments or refinements were made to the overall Pain P/P in-order to integrate into routine processes and/or practices throughout hospital after 2007? *This may differ by service, program or by unit etc.* (I.e. even between medicine care units because it may be based on daily routines, org structure and MD rounds etc.)   2. Are they applying models of continual refinement that will support impact of the Pain P/P in practice?   3. What is the long term plan to commit resources for training and ongoing adaption for the Intervention?   4. Who has been involved in adapting cpnts of the Pain P/P to better fit within your hospital level?   5. Who has been involved in adapting cpnts of the Pain P/P to better fit at the unit level?   6. How are they monitoring the need for refinements? (E.g. Steering Cttee and champions, biannual prevalence training, audit & Feedback process commitments).   7. Which of the 5 target behs do they feel are important and why?   8. What target behs still need work to integrate into routine practices at the hospital, dept. or service levels? |
| **DSF TENET/**  **PROPOSITION** | **DSF Label** | **Short User friendly explanation** | **Specific Example** | **Application to this Case Study**  **Phase of Study**  **Data Sources** | **Draft Questions** |
| 3. Ongoing feedback on HC Intervention needs to use practical, relevant measures of progress and relevance. | 3. **Ongoing Feedback** is necessary for sustainment of Pain P/P | Refers to the measures they are using to monitor if people are using the Pain P/P | Look for practice surveys, audits, monitoring tools communication tools etc. measuring progress related to the ongoing use of the Pain P/P | Phase I =identify measures with  Document review,  Examine the types of measures, structures, processes being used to see if they are...  - relevant to desired outcomes of pts,  - sensitive to the fit btwn the Intervention and the context and  -are feasible to sustain  *Confirm if changes to prevalence audits and pt. surveys overtime are based on results and focus set by the Steering cttee biannually.* | **Ongoing Feedback- Department Level**   1. What measures are they using to provide feedback on nurses ongoing implementation of the 5 target behs? 2. What processes are in place to measure policy use at the department level? 3. What structures are in place to measure policy use at the department level? 4. Do they find the pt. satisfaction reports helpful to maintain the use of the Pain p/P or 5 target behs? 5. What do they do with reports, audit results, survey results related to Pain P/P ongoing use? |
| **DSF TENET/**  **PROPOSITION** | **DSF Label** | **Short User friendly explanation** | **Specific Example** | **Application to this Case Study**  **Phase of Study**  **Data Sources** | **Draft Questions** |
| 4.Voltage drop is not inevitable within a culture of Continuous Quality Improvement (CQI)  *Definition:*  *Voltage drop = assumes the more diverse and complex a patient pop is, the smaller the benefit of the HC Intervention.* | **Voltage** must be **maintained** or possibly **increased overtime** for sustainment**.** | Suggests a culture of improvement is central to the ongoing use of the Pain P/P and the sustainment process | Refers to whether people are (🡩or🡫) their use of Pain P/P overtime | Phase I examine usage overtime via document review  Phase II – confirm usage overtime and presence of a culture of ongoing CQI to be better able to adapt the Pain P/P to contexts and pts. via ...  interviews,  Unit specific document review. | **Department Culture & Voltage overtime**   1. Do you feel the org maintains a culture of ongoing improvement? 2. If YES or NO ….Please explain how the culture has influenced the use of the 5 target behs within the hospital? 3. What factors if any could influence a culture of improvement in your organization and its use of the 5 target behs   **Unit Culture & Voltage overtime**   1. What type of culture do you feel the unit has? 2. Do you feel the unit maintains a culture of ongoing improvement? 3. If YES or NO ….Please explain how the culture has influenced the use of the 5 target behs on your unit? 4. What other factors if any could influence a culture of improvement on your unit and its use of the 5 target behs? |
| **DSF TENET/**  **PROPOSITION** | **DSF Label** | **Short User friendly explanation** | **Specific Example** | **Application to this Case Study**  **Phase of Study**  **Data Sources** | **Draft Questions** |
| 5. Sustainment of an HC Int (Pain P/P) will be maintained when there is a ‘strong fit’ btwn the Intervention and the context | **Strategies** and **Factors** influencing **Fit**  Multi-level aspect here ...apply only  to unit level here | Strong fit btwn Pain P/P & context is need for sustainment | They are surveying target behs & providing unit specific feedback to managers in a timely manner (1-month post audit) to develop remedial action plans to 🡩 adherence rates | Phase II identify strategies via... unit specific document review, and Interviews  *Look for evidence of how they are obtaining fit = adaption of Pain P/P to the unit level to sustain it.* | **Unit Level Strategies & Factors influencing Fit**   1. What strategies have been helpful *to you* *personally* to maintain your ongoing use of the 5 target behs? 2. What strategies have been helpful to *your unit* as a whole to sustain the use of the 5 target behs? 3. What other type of information or (measurements) would you find helpful to maintain your use of the Pain P/P or 5 target behs from here on? 4. What factors if any have influenced your ongoing use of the 5 target behaviors on your unit? 5. What factors have influenced the unit as a whole to continue to use the 5 recommendations? |
| **DSF TENET/**  **PROPOSITION** | **DSF Label** | **Short User friendly explanation** | **Specific Example** | **Application to this Case Study**  **Phase of Study**  **Data Sources** | **Draft Questions** |
| 6.Organizational Learning should be a core value of the implementation setting | 6. **Learning Org is a Core Value** for sustainability | Focuses on development of problem-solving capacity at multiple levels. | Look for evidence of problem solving strategies at corporate and unit level to further develop, deliver and sustain Pain P/P. | Phase I –identify problem solving capacity at department level via...  document review  Phase II – identify problem solving activities to sustain Pain P/P at unit level via...  document review,  interviews | **Learning Org capacity-Department level**   1. What department level processes, structures, resources have been established to sustain the Pain P/P? 2. What is the department problem solving approach?   **At Unit Level**   1. What is the unit’s problem solving approach? 2. Has the unit used this approach to integrate any of the 5 target behs into routine practice? 3. If YES what has worked? 4. If NO …Why has it not worked or been used? |
| **DSF TENET/**  **PROPOSITION** | **DSF Label** | **Short User friendly explanation** | **Specific Example** | **Application to this Case Study**  **Phase of Study**  **Data Sources** | **Draft Questions** |
| 7. Ongoing stakeholder involvement throughout should lead to better sustainability | **Ongoing stakeholder involvement** is necessary for sustainability | Shared visioning and engagement of stakeholders from all levels within the org are necessary for department wide sustainment of Pain P/P. | Department level=  Shared visioning & planning with Interprofessional grps in policy chgs, training of staff for biannual audits, MD involvement etc.  Unit level=  Staffing changes,  policy adaptions to unit practices,  engagement in ongoing remedial actions rt biannual audit results | Phase I- Identify stakeholders department and corporate level via...  Document review  Interviews.    Phase II – identify stakeholders via...  unit specific document review,  Chart audit (use of consultants /experts),  Interviews  *Look for evidence of engagement with internal and external stakeholders in “planning, implementation, adaptation of processes, to help increase the fit btwn the local context, and help address evolving issues (barriers) that interfere with its ongoing use.* | **Department Level Stakeholders**   1. Who were the stakeholders between 0-2 yrs. ? 2. Who are the stakeholders for 2yrs to present?   **Unit Level Stakeholders**   1. What involvement have you had on the ongoing implementation of the Pain P/P on your unit? Ie planning, processes, adaption of policy, embedding into routine practices? 2. Who are the stakeholders involved in the ongoing implementation of the Pain P/P on your unit? 3. Is there anyone you think should be involved but is not? 4. If so who, why and how? |

**References**

1. Chambers DAG, R. E.;Stange, K. C. The dynamic sustainability framework: addressing the paradox of sustainment amid ongoing change. Implementation Science. 2013;8:117.
