# Supplementary material for: Sustaining a nursing best practice guideline in an acute care setting over 10 years: A mixed methods case study
Source: Front Health Serv. 2022 Aug 30;2:940936. doi: 10.3389/frhs.2022.940936 (PMC10012662; doi:10.3389/frhs.2022.940936)
Supplement: Supplementary file 3 [file Table_3.docx]

**Supplementary material 3.** Data sources and measures

| Report Number | Title and Dates created or issued |
| --- | --- |
| Rt1 | Notes from meeting on monitoring and auditing results with NPP Coordinator 2015 |
| Rt2 | Teleconference minutes with APN for acute care Nov 18, 2015 |
| Rt3 | List of Corporate and Unit level KTIs implemented 2006-2017: summary of strategies |
| Rt4 | Notes during meeting with NPP Coordinator March 31, 2015 |
| Rt5 | Notes during meeting with APN Palliative Care -Lead for BPG project initial start-up Oct 20, 2015 |
| Rt6 | Minutes from Meeting with NPP Coordinator May 27, 2016 |
| Rt7 | Table of prevalence data measures for Pain PP target behaviours and ELM records performance measurement improvements 2011-2015 |

| Internal Document Number | Title and Dates issued |
| --- | --- |
| ID1 | 2015 EBP Implementation power point presentation by NPP |
| ID2 | NPP strategic plan on adherence to Pain P/P  Multiple plans -2005/2006, 2007/2008, 2008/2009, 2009/2010, 2010/2011, 2011,2012, 2012/2013, 2013/2014, 2014/2015, 2015/2016 |
| ID3 | Terms of Reference Pain Assessment and Management BPG work Group 2006 |
| ID4 | 10 themes used for Pain P/P development |
| ID5 | 2007 Pain Assessment and Management Policy |
| ID6 | 2013 Pain Assessment and Management Policy |
| ID7 | Pain Prevalence Audit Tool= Patient Assessment questions & Chart audit questions Nov 2010 |
| ID8 | Pain Prevalence Audit Tool= Patient Assessment questions & Chart audit questions Nov 2012 |
| ID9 | Pain Prevalence Audit Tool= Patient Assessment questions & Chart audit questions Nov 2013 |
| ID10 | Pain Prevalence Audit Tool= Patient Assessment questions & Chart audit questions April 2014 |
| ID11 | Pain Prevalence Audit Tool= Patient Assessment questions & Chart audit questions April 2015 and Nov 2015 |
| ID12 | Post Discharge Patient Satisfaction Survey tool |
| ID13 | Site Final Report for BPSO on RNAO Pain Assessment and Management BPG implementation Oct 2011 by CB |
| ID14 | Hourly Rounding Policy versions 2012, 2014 |
| ID15 | Brief Pain Inventory -Self Report 2005 |
| ID16 | 24 Nursing Documentation Flowsheet: versions 2008, 2014,2016 |
| ID17 | Patient Teaching Record 2009 |
| ID18 | Patient Admission History: versions 2013, 2016 |
| ID19 | Patient Assessment and Medication Administration Record 2009 |
| ID20 | Provisions of Additional Therapy Services by External Providers 2014 |
| External Document Number | Title and Dates issued |
| ED1 | RNAO Pain Assessment and management BPG 2007 |
| ED2 | RNAO Pain Assessment and management BPG 2013 |

**Key**

Reports (Rt#)

Internal documents (ID#)

External documents (ED#)

List of KTIs used for Pain P/P over time 2005-2017

| **Pain Management - Knowledge Translation Interventions (KTI) listing** | | | | |  |
| --- | --- | --- | --- | --- | --- |
| **Date** | **Target Group** | **KTI Activity** | | | |
|  |  | **Policy/Guideline/Tools & Form development** | **Training** | **Infrastructure support** | **Auditing/ quality performance monitoring** |
| 2005 +ongoing | Corporate wide | Introduction to Brief Pain Inventory (BPI) |  |  |  |
| 2005+ongoing | Corporate wide | Assmt Guidelines for Infusions used for Pain mgmt |  |  |  |
| 2005 | Corporate wide | Acute Pain Mgmt Policies developed (Epidural, IV PCA, Regional Analgesia, Single Dose Intrathecal) |  |  |  |
|  |  |  |  |  |  |
| 2006 | Corporate wide |  | Corporate Nursing Orientation |  |  |
| 2006 | Corporate wide |  |  | Pain Council |  |
| 2006 + ongoing | Corporate wide |  | Pain Education Days (offered x 2 per year ) |  |  |
| 2006 | Corporate wide |  | Best Practice Champions (60 trained) |  |  |
|  |  |  |  |  |  |
| 2007 | Corporate wide |  |  | Pain Council |  |
| 2007 | Corporate wide | Pain Assmt & Mgmt Corporate Policy developed |  |  |  |
| 2007 Oct | Corporate wide |  | Pain Awareness Week (education initiative) |  |  |
| 2007 + ongoing | Corporate wide |  | Pain Education Days (offered x 2 per year) |  |  |
|  |  |  |  |  |  |
| 2008 | Corporate wide |  |  | Pain Council |  |
| 2008 | Corporate wide | Pain Assmt & Medication Admin Record for Infusions used for Pain Mgmt |  |  |  |
| 2008 +ongoing | Corporate wide  Outpt Oncology Clinics | Introduction of ESAS (Self-reporting symptom Mgmt screening tool) includes pain in Outpt Oncology clinics. |  |  |  |
| ???? + ongoing | Corporate wide  Outpt Oncology Clinics | Online ESAS Tool (Self-reporting symptom Mgmt screening tool) includes pain in Outpt Oncology clinics. Monitoring & Reporting to Cancer Care Ontario |  |  |  |
| 2008 Oct | Corporate wide |  | Pain Awareness Week (education initiative) |  |  |
| 2008 + ongoing | Corporate wide |  | Pain Education Days (offered x 2 per year) |  |  |
|  |  |  |  |  |  |
| 2009 – 2014 | Corporate wide |  |  | Pain Council |  |
| 2009 | Corporate wide | Dosing of Opioids for Acute Pain in Opioid naïve patients |  |  |  |
| 2009 Oct | Corporate wide |  | Pain Awareness Week (education initiative) |  |  |
| 2009 + ongoing | Corporate wide |  | Pain Education Days (offered x 2 per year) |  |  |
| 2009-2010 | Corporate wide |  |  |  | Development of electronic software for prevalence auditing |
|  |  |  |  |  |  |
| 2010 - 2014 | Corporate wide |  |  | Pain Council |  |
| 2010 Oct | Corporate wide |  | Pain Awareness Week (education initiative) |  |  |
| 2010 + ongoing | Corporate wide |  | Pain Education Days (offered x 2 per year) |  |  |
| Nov 2010 | Corporate wide |  | Prevalence training and survey |  |  |
|  |  |  |  |  |  |
| 2011 | Corporate wide |  |  | Pain Council |  |
| 2011 Oct | Corporate wide |  | Pain Awareness Week (education initiative) |  |  |
| 2011 | ? | Patient and Family Member Information Guide: Pain Assmt & Mgmt |  |  |  |
| 2011 | ? | Patient and Family Member Information Guide: Pain Mgmt After Surgery |  |  |  |
| 2011 + ongoing | Corporate wide |  | Pain Education Days (offered x 2 per year) |  |  |
| 2011 + ongoing | Oncology |  | Oncology nursing orientation 1/2 day on pain management |  |  |
| Nov 2011 | Corporate wide |  | Prevalence training and survey |  |  |
|  |  |  |  |  |  |
| 2012 -2014 | Corporate wide |  |  |  | Corporate Scorecard - pain satisfaction |
| 2012 -2014 | Corporate wide |  |  |  | Manager - performance goals |
| 2012- 2014 | Corporate wide |  |  | Pain Council |  |
| 2012 | Corporate wide |  | Pain eLearning modules - mandatory training |  |  |
| 2012 + ongoing | Palliative |  | Palliative Care education days with one half day on pain - offered twice per year |  |  |
| 2012 + ongoing | Corporate wide |  | Pain Education Days (offered x 2 per year) |  |  |
| 2012 + ongoing | Oncology |  | Oncology nursing orientation 1/2 day on pain management |  |  |
| Apr 2012 | Corporate wide |  |  |  | Prevalence training and survey |
| Nov 2012 | Corporate wide |  |  |  | Prevalence training and survey |
|  |  |  |  |  |  |
| 2013 -2014 | Corporate wide |  |  |  | Corporate Scorecard - pain satisfaction |
| 2013 -2014 | Corporate wide |  |  |  | Manager - performance goals |
| 2013 - 2014 | Corporate wide |  |  | Pain Council |  |
| 2013 | Corporate wide | Hourly rounding/Bedside shift report/care boards= whiteboard |  |  |  |
| 2013 | Corporate wide | Pain Assmt & Mgmt Corporate Policy revised |  |  |  |
| 2013 + ongoing | Palliative |  | Palliative Care education days with one half day on pain - offered twice per year |  |  |
| 2013 + ongoing | Corporate wide |  | Pain Education Days (offered x 2 per year) |  |  |
| 2013 + ongoing | Oncology |  | Oncology nursing orientation 1/2 day on pain management |  |  |
| Apr 2013 | Corporate wide |  |  |  | Prevalence training and survey |
| Nov 2013 | Corporate wide |  |  |  | Prevalence training and survey |
|  |  |  |  |  |  |
| 2014 | Corporate wide |  |  |  | Corporate Scorecard - pain satisfaction |
| 2014 | Corporate wide |  |  |  | Manager - performance goals |
| 2014 | Corporate wide |  |  | Pain Council |  |
| 2014 | Corporate wide | Nurse Leader Rounding |  |  |  |
| 2014 + ongoing | Palliative |  | Palliative Care education days with one half day on pain - offered twice per year |  |  |
| 2014 + ongoing | Corporate wide |  | Pain Education Days (offered x 2 per year) |  |  |
| 2014 + ongoing | Oncology |  | Oncology nursing orientation 1/2 day on pain management |  |  |
| Apr 2014 | Corporate wide |  |  |  | Prevalence training and survey |
| Nov 2014 | Corporate wide |  |  |  | Prevalence training and survey |
|  |  |  |  |  |  |
| 2015 + ongoing | Palliative |  | Palliative Care education days with one half day on pain - offered twice per year |  |  |
| 2015 + ongoing | Corporate wide |  | Pain Education Days (offered x 2 per year) |  |  |
| 2015 + ongoing | Oncology |  | Oncology nursing orientation 1/2 day on pain management |  |  |
| 2015 + ongoing | Oncology |  | LEAP Mini Oncology - interprofessional day on Palliative Care including pain |  |  |
| Apr 2015 | Corporate wide |  |  |  | Prevalence training and survey |
| Nov 2015 | Corporate wide |  |  |  | Prevalence training and survey |
|  |  |  |  |  |  |
| 2016 + ongoing | Palliative |  | Palliative Care education days with one half day on pain - offered twice per year |  |  |
| 2016 + ongoing | Corporate wide |  | Pain Education Days (offered x 2 per year) |  |  |
| 2016 + ongoing | Oncology |  | Oncology nursing orientation 1/2 day on pain management |  |  |
| 2016 + ongoing | Oncology |  | LEAP Mini Oncology - interprofessional day on Palliative Care including pain |  |  |
| Apr 2016 | Corporate wide |  |  |  | Prevalence training and survey |
| Nov 2016 | Corporate wide |  |  |  | Prevalence training and survey |
|  |  |  |  |  |  |
| Ongoing | Corporate wide |  |  | NPPC strategic plans,  minutes of the pain workgroup,  Pain Council minutes |  |
| Aug 2016 | Medicine Portfolio |  | Medicine (A5/B5) education by Acute Pain APN and Palliative APN |  |  |
| Aug 2016 | Corporate wide | Inclusion of pain scores on nursing flow sheet |  |  |  |
|  |  |  |  |  |  |
| 2017 + ongoing | Palliative |  | Palliative Care education days with one half day on pain - offered twice per year |  |  |
| 2017 + ongoing | Corporate wide |  | Pain Education Days (offered x 2 per year) |  |  |
| 2017 + ongoing | Oncology |  | Oncology nursing orientation 1/2 day on pain management |  |  |
| 2017 + ongoing | Oncology |  | LEAP Mini Oncology - interprofessional day on Palliative Care including pain |  |  |
| Apr 2017 | Corporate wide | Prevalence training and survey |  |  | Prevalence training and survey |
| Nov 2017 | Corporate wide | Prevalence training and survey (champions =170) |  |  | Prevalence training and survey (champions=170) |

Biannual prevalence survey measures and education training records mapped to the Pain P_P

| **Pain P/P Target Behavior** | **Data Sources = CA (Chart Audit) or PA (Pt Assessment)** | **Nov. 2010** | **Apr. 2011** | **Nov. 2011** | **Apr. 2012** | **Nov. 2012** | **Apr. 2013** | **Nov. 2013** | **Apr. 2014** | **Nov. 2014** | **Apr. 2015** | **Nov. 2015** | **Comments** |
| --- | --- | --- | --- | --- | --- | --- | --- | --- | --- | --- | --- | --- | --- |
| **#1 on admission** | CA= Q1 (Nov 2010-Apr 2012) | x | x | x | x |  |  |  |  |  |  |  | 4 CA data points |
| **#2 once per shift -documented ongoing pain reassessment within last 24 hrs** | CA= Q3 (2010 - Apr 2012) | x | x | x | x |  |  |  |  |  |  |  | 4 CA data points |
| **#2 once per shift** | CA =Q11 (2012-Nov 2015) |  |  |  |  | x | x | x | x | x | x | x | 7 CA data points |
| **#2 once per shift** | PA Q2 (2013 to 2015) |  |  |  |  | x | x | x | x | x | x | x | 6 PA data points |
| **#3 Documented pain during stay** | CA = Q1 (2013 to 2015) |  |  |  |  |  | x | x | x | x | x | x | 6 CA data points |
|  | PA Q2 & PA Q3 (2010), | x | x | x | x |  |  |  |  |  |  |  | 4 PA data points |
|  | PA Q8 (2012),  PA Q1 (2013 to 2015) |  |  |  |  | x | x | x | x | x | x | x | 7 PA data points |
| **#4 documented pain during hourly rounding.** | Nil |  |  |  |  |  |  |  |  |  |  |  | no data points |
| **#5 Intensity** | CA = Q2 (Nov 2010 to Apr 2012) | x | x | x | x |  |  |  |  |  |  |  | 4 CA data points |
| **gives numeric value for pain 0-10** | PA Q9 (Nov 2012) and PA Q1a (2013 to 2015) |  |  |  | x | x | x | x | x | x | x | x | 8 PA data points |
| **#6 Pain Goal established** | CA = Q5 (Nov 2010 only) | x |  |  |  |  |  |  |  |  |  |  | 1 CA data point |
|  | PA Q5 (Apr 2014 to 2015) |  |  |  |  |  |  |  | x | x | x | x | 4 PA data points |
| **#7 Collaborates with pt on pain strategies** | CA Q5 (Nov 2010) only | x |  |  |  |  |  |  |  |  |  |  | 1 CA data point |
| **# 8 Reassess pain intensity** | PA Q12 (Nov 2012) PA Q4 (Nov 2013 to 2015) |  |  |  |  | x | x | x | x | x | x | x | 7 PA data points |
| **# 9 Assess effect of pharmacological interventions** | PA Q7 (Nov 2010)  PA Q10.2 (Nov 2012)  PA Q2a (Nov 2013) PA Q2b (Nov 2014 & 2015) | x | x | x | x | x | x | x | x | x | x | x | 11 PA data points |
|  | PA Q8 (2010) only | x |  |  |  |  |  |  |  |  |  |  | 4 PA data points |
|  | PA Q11 (2012) PA Q3 (2013 to 2015) |  |  |  |  | x | x | x | x | x | x | x | 7 PA data points |
| **#10 Monitor side effects** | nil |  |  |  |  |  |  |  |  |  |  |  | no data points |
| **#11 Consults with experts** | CA = Q6 (Nov 2010 only) | x |  |  |  |  |  |  |  |  |  |  | 1 CA data points |
| **# 12 Educate Pt & Family** | CA = Q7 (Nov 2010),  CA = Q12 (Nov 2012) | x | x | x | x | x | x |  |  |  |  |  | 6 CA data points |
|  | PA 4 (Nov 2010) | x |  |  |  |  |  |  |  |  |  |  | 1 PA data point |
| **#13 Ongoing document to comm with IP team** | nil |  |  |  |  |  |  |  |  |  |  |  | no data points |
| **#14 Ongoing Edu of staff** | ELM records (Nov 2011-Nov 015) |  |  | x | x | x | x | x | x | x | x | x | 9 data points |

**Key:** CA = chart Audit, PA = Patient Assessment conducted during audit, Q# = Question number (#) on audit form/tool, Pt = Patient, Edu = Education, IP = Inter Professional
