# Supplementary material for: Sustaining a nursing best practice guideline in an acute care setting over 10 years: A mixed methods case study
Source: Front Health Serv. 2022 Aug 30;2:940936. doi: 10.3389/frhs.2022.940936 (PMC10012662; doi:10.3389/frhs.2022.940936)
Supplement: Supplementary file 4 [file Table_4.docx]

**Supplementary material 4**: Department nurse determinants, KTIs, and documents mapped to DSF constructs (1)

| **DSF Constructs**  **Innovation**  **Practice Setting**  **Broader system** | **Implementation Determinants**  **(0-2 yrs.) (3)** | **Sustainability**  **Determinants (12)**  **(>2-10 yrs.)** | **Documents** | **Implementation**  **KTIs (12)**  **(0-2 yrs.)** | **Sustainability**  **KTIs (21)**  **(>2-10 yrs.)** | **Document** |
| --- | --- | --- | --- | --- | --- | --- |
| **DSF Innovation/Intervention Construct (below)** | | | | | | |
| Innovation components Characteristics |  |  |  | **֍ Embedding of** **Pain P/P** into existing unit processes (P2,P3) | **֍ Embed ongoing refinements** into existing routine practices/processes & Pain P/P (P1,P2,P3) | **ID5-6, ID14-20, ID2, Rt4, Rt5, Rt6** |
| Delivery platform |  |  |  |  |  |  |
| Outcomes =Effectiveness of innovation for patient, staff, org |  |  |  |  |  |  |
| innovation practitioners (set of characteristics defining who should deliver |  |  |  | Pain P/P established **Interdisciplinary** for all disciplines (P1,P2,P3) |  | **ID1, ID3, ID5, ID20** |
|  |  | **Facilitator (P1,P2,P3)**  Positive user attitudes and values related to the use of the Pain P/P | **ID1, ID3, Rt2, Rt4** |  |  |  |
| **Practice Setting (Context) Construct (below)** | | | | | | |
| Staffing =Human resources & capital resources exists within the practice setting |  |  |  | **Joint collaboration** of human resources **from all levels of nursing** plus other disciplines to develop departmental implementation plan (P1,P2,P3) |  | **ID2, ID4, Rt4-6, ID13** |
|  |  |  |  | **Secure internal financial** commitment – time and Human resources to participate on Cttees and to implement KTIs (P1,P2,P3) |  | **ID1-2, Rt3-4, Rt6** |
|  |  | **Barrier (P1,P2)**  bimodal staff complement | **Rt2, Rt4** |  |  |  |
|  |  | **Barrier (P2)**  Turnover of students(medical) | **ID13** |  |  |  |
| supervision | **Facilitator (P1,P2,P3)**  ***** Leadership Commitment** (NPP) | **Facilitator (P1,P2)**  ***** Formal Leadership support at dept.** (CNO) and **unit level (**Clinical leaders, Educators & Champions) | **ID1, Rt2, Rt3, Rt4, Rt5, Rt6, ID13** |  |  |  |
|  |  |  |  | **֍ Formalize BPG Coordinator role** to lead ongoing implementation within NPP dept.(P2,P3) | **֍** NPP **comparing survey results among units created a sense of competition** among leaders and users to improve (P1,P2) | **ID1, Rt4, Rt6** |
| Organization  Culture /climate |  | **Barrier (P1,P2,P3)**  Competing corporate priorities | **ID2, Rt3, Rt5,**  **ID7, ID11** |  | **Dept. determine EBP priorities (P1,P2,P3)** | **ID2-3, Rt3, Rt7** |
| Training processes |  |  | **ID2, Rt3, Rt5,**  **ID7, ID11** | use **multi-modal approach to disseminate** **(P1,P2,P3)** |  | **ID1, Rt4-5, ID13** |
|  |  |  |  |  | **Ongoing pain care education** support at dept. and unit levels becomes tailored over time i.e. 1 on 1 , case studies **(P1,P2,P3)** | **ID1-2, Rt3, Rt5-7** |
|  |  |  |  |  | Develop unit specific **additional resources/tools (P1,P2,P3)** | **ID2-3, Rt3, Rt5** |
|  |  |  |  |  | Mandatory **eLearn training** system (P1,P3) | **ID2, Rt3, Rt6, Rt7** |
|  |  |  |  | **֍ Pain Council established - Interdisciplinary** **taskforce** leads initial policy development, education strategies and future policy revision (P1,P2,P3) | **֍** **NPP reps develop formal and informal education** initiatives at dept. and unit level in 2014 initially performed by the Pain Council. (P1,P2,P3) | **ID2, Rt3, Rt6** |
|  |  |  |  | **֍ Educating Champions** – Education of 60 to be clinical experts on units, with APNs (P2,P3) | **֍**  Provides **Unit level expertise** to support use of Pain P/P a total of 170 experts = Champions, educators, APNs, work across units as clinical resource (P1,P2,P3) | **ID1, ID4, Rt4** |
|  |  |  |  |  | Ongoing biannual **training** of staff **to conduct prevalence survey (**P1,P2,P3) | **Rt3, ID13** |
|  |  |  |  |  | **Unit specific training** of staff provided **based on audit remedial action plans** to improve on related BPG survey indicators (P1,P2,P3) | **Rt3, Rt5, Rt6** |
| Information systems= org communication capacity for monitoring (exchange and feedback) |  |  |  |  | **NPP Establishes regular performance monitoring:** includes results from biannual prevalence audit and internal incident reporting (P1,P2,P3) | **ID1, Rt3, Rt6** |
|  |  |  |  |  | Timely exchange of prevalence survey results led to **course correcting changes** (P1,P2,P3) | **Rt5, Rt7, ID7-11** |
|  |  |  |  |  | **֍ Development of an** **electronic monitoring system** to measure nursing sensitive indicators provide monitoring of BPG adherence (aligns with 14 Imp b) (P1,P2) | **ID2, Rt1, Rt4-7** |
| business model structure & system to monitor/manage innovation |  |  |  | **֍ Obtaining buy-in and Formalize nurse leaders’ involvement** on Steering Cttee. (P1,P2,P3) | **֍ Corporate level Internal Cttees’ support ongoing review of clinical tactics** support sustained use ie Patient Experience Steering cttee and Accreditation workgroup.(P2) | **ID1, ID3** |
|  |  |  |  | **֍ Established Pain BPG taskforce/workgroup** in NPP dept. – enduring central reporting and monitoring structure for ongoing implementation and evaluation (P1,P2,P3) | **֍ NPP and Unit Leaders facilitate/lead remedial action plan for under performing units (P1,P2,**P3) | **ID1-3, Rt3-5, ID13** |
|  |  |  |  |  | **Unit leaders lead dept. and unit level patient centered initiatives for pain care based on unit routine practices** -with adoption of EBP care | **ID2, Rt3** |
|  |  |  |  |  | **Performance Evaluation indicators** for monitoring rt innovation = Mangers, + staff (P1,P2,P3) | **ID1, Rt1, Rt4, Rt7, ID7-11** |
|  |  |  |  |  | **Spread EBP** to additional areas (P1.P2.P3) | **Rt1,Rt3,Rt4** |
| New DSF Factor |  | **Facilitator (P1,P2,P3)**  **Board of Directors & VP leadership corporate level commitment** to EBP as a shared priority | **ID1, ID2, ID13** |  |  |  |
| **Broader System construct (below)** | | | | | | |
| Market forces | **Facilitator (P1, P2, P3)**  *******Timely call from RNAO for BPSO applications | **Facilitator** (P1,P2,P3)  ******* New updated **version release of BPG** from RNAO-agency | **ID1, ID2, ID3, ID5, ID6, ED1, ED2** |  |  |  |
|  |  | **Facilitator**(P1,P2,P3)  **National and International releases** focused on innovation | **ID1, Rt5** |  | **Staff participation on a regional network- -** provide access to new research and related outcomes for pain mgmt ( P1,P2,P3) | **ID3, ID6, ED2** |
|  |  |  |  |  | Integrating **new research/evidence** released into BPG and ongoing education(P1,P2,P3) | **ID1-3, ID5-6-, ED1-2** |
| Other practice settings |  |  |  |  |  |  |
|  |  | **Facilitator (P1,P2,P3)**  **Goal alignment** with Education Inst – Facilitator + E1 Education Institution support for innovation in support for EBP | **ID13** |  |  |  |
|  |  | **Facilitator**(P1,P2,P3)  **Formal External recognition** by related BPG association | **ID1, Rt5** |  |  |  |
|  |  |  |  |  | **Benchmarking** to external sources best practices (P1,P2,P3) | **Rt4, Rt6, ID13, ED1-2** |
| Population characteristic’s | **Facilitator (P1, P2, P3)**  *** **Need** to improve (P1, P2) or standardize from Pt satisfaction reports (P2, P3) | **Facilitator** (P1,P2,P3)  *******Increasing **Consumer demand for pain management** | **ID4, Rt1, ID13** |  |  |  |
| legislative environment /regulation |  | **Facilitator**(P1,P2,P3)  **External pressure** from accrediting agencies & ministry | **Rt4** |  |  |  |
| policy |  |  |  |  |  |  |
| New DSF Factor |  |  |  | **Use frameworks** to guide implementation and Id barriers (P1, P2) |  | **Rt5, ID13** |
| New DSF Factor |  |  |  | ***Secure external funds (P1,P2,P3)**   1. **RNAO PBSO –** secure **operating** funds for initial training and resource s to build capacity **(P1,P2)** 2. **֍** CNF- secure **capital** external financial support - for point of care surveying system (P2,P3) |  | **ID1, ID13** |

**Key**:

P# = Participant code, ID# = Internal Document code, ED# = External Document code, Rt# = Report number (document type), DSF = Dynamic Sustainability Framework

******* = Determinants that had a continuous influence in both the implementation and sustained use phases over time

**֍ =** KTIs that had a continuous influence in both the implementation and sustained use phases over time

**References**

1. Chambers DAG, R. E.;Stange, K. C. The dynamic sustainability framework: addressing the paradox of sustainment amid ongoing change. Implementation Science. 2013;8:117.
