# Supplementary material for: Sustaining a nursing best practice guideline in an acute care setting over 10 years: A mixed methods case study
Source: Front Health Serv. 2022 Aug 30;2:940936. doi: 10.3389/frhs.2022.940936 (PMC10012662; doi:10.3389/frhs.2022.940936)
Supplement: Supplementary file 5 [file Table_5.docx]

**Supplementary material 5a.** Unit nurses’ determinants, quotes, and documents mapped to DSF constructs (1)

|  |  |  | **Facilitators** | | **Barriers** | |  |  |
| --- | --- | --- | --- | --- | --- | --- | --- | --- |
| **DSF**  **Determinants** | **Determinant**  **Code** |  | **Subcase 1  N=8**  **(C1-8)** | **Subcase 2  N=8**  **(G1-8)** | **Subcase 1  N=8**  **(C1-8)** | **Subcase 2  N=8**  **(G1-8)** | **Quotes** | **Documents** |
| **DSF Innovation/Intervention Construct** | | | | | | | | |
| Innovation outcome effectiveness | Family /Pt Preferences |  |  |  | N=3 C3, C6, C7 | N=5  G1, G2, G3, G4, G8 | "Family and patient preferences for opioid medications is a factor" (C3,C7)  "Pts with addictions have preferences for +++opioids or they come in and they’re on IV or sub-q Dilaudid then it’s time for them to go home so we switch them to a pill and then they’re not happy about that as well...so you know it’s all these different situations where it’s very difficult to not adhere to the pain policy but kind of take it in stride" (C2-P3) |  |
| Innov practitioners defining characteristics | MD Prescribing Preferences |  |  |  | N= 5 C3, C4,C5, C6, C7 | N=4  G1, G3, G6, G8 | "the Medicine doctors are very reluctant to give pain medication sometimes...if something is not effective, and you’re saying you know this isn’t really working, I’ve gotten a lot of like push back, they might eventually do it but if they’re not really familiar with the patient they’re really hesitant" (C1-P4).  "new residents are a little skittish around narcotics , sometimes that a barrier and they order a lot less than you need" (C2-P3), or you are waiting for the order and its kind of like chasing them (MDs) (C2-P6) |  |
|  | Nurses Motivation to use Pain P/P |  | N=4 C3,C6,C7,C8 | N=5 G1,G3,G5,G6,G7 |  |  | "They (*Nurses*) accept it (pain policy) 100%. I think it’s like I said, I think it is ingrained in us and it has been ingrained in us for a long time" C2-P1; "Nurses are very supportive of the use of evidence--based practices such as the Pain P/P "(C1-P3); "I think people are very one with the policy, I think that pain management is a big deal here or a concern for everybody for sure" (C1-P7).  "I'm using it (*Pain P/P)* all the time “(C2-P3);” I'm front line right? I am the one interacting. Ultimately, I think that I can make the most difference ... noticing if my patient’s in pain, advocating for my patient, making sure I’m following through with the PRNs and everything" (C2-P4); " I guess just actively like using it (*Pain P/P*), like making sure that I follow the policy and reviewing it, making sure that I’ve read this policy" (C2-P6). |  |
|  | Supportive IP Team members |  | N=2 C2,C6 | N=3 G3,G6,G8 |  |  | "...we have a very supportive IP care team working to manage pain and we’re really open to using other services to help us out with pain care and not just medications" C1-P2).   "it’s a very family dynamic style on the unit, everyone's very close. If someone sees you drowning, they’re really good at stepping up and trying to take on your load even if it’s just you know, a quick check in on your patients to see if they’re okay. "(C2-P8), "we are good at communicating and collaborating with each other on pain issues" (C2-P6) |  |
|  | Senior nurse resistance |  |  |  |  | N=2 G7,G8 | " senior nurses are stuck in the older school nursing and rarely use alternative therapies"(C2-P8) …and "Some nurse are stuck in their ways and new nurses are more likely to follow policy "( C2-P7) |  |
|  | Nurses experience/expertise |  |  | N=2 G3,G8 |  |  | "...more experience nurses also help out a lot, because you know, the questions you ask them they know the answers" (C2-P3). |  |
| **DSF Practice Setting (Context) Construct** | | | | | | | | |
| Staffing =Human resources & capital resources exists within the practice setting | Supportive Multiple stakeholders |  | N=8 C1,C2,C3,C4,C5,C6,C7,C8 | N=8 G1,G2,G3,G4,G5,G6 ,G7,G8 |  |  | Key stakeholders influencing Pain P/P use are: "**frontline nurses** do assessments, refer to MDs, consult and advocate for patients" (C1-P2, P4 -8); "nurse **educator** is very involved and helpful" (C1-P1, P3-8); "**MDs** do as well" (C1-P2, P5-6); "we have acute pain service experts for people who have difficult situations with pain" (C1-P1, P4-5, P7); "**patient, /family** get involved" (c1-P2, P4,,P7-8); "our manager brings feedback information to use regarding biannual survey result" C1-(P4,P8); "we use **palliative care experts** mainly for palliative patients" (C1-1,P7); " **pharmacists** do review of medications and answer questions you have" (C1-P8); "We always reach out to **Physiotherapists** whenever there is some king of mobility or if patients have difficulty mobilizing" (C1-P2).   "**Nurses** model use of pain P/P " (C2-P1, P5); "we rely on our senior nurses a lot" (C2-P3); "**doctors** are consulted by nurses when they can't control the patient's pain" (C2-P8); "if I had any questions about the policy it’s our **educator** that I would go to, and she’s good at like taking time with us, reviewing it, and answering our questions" (C2-P6); "the **palliative care team** are amazing for chronic pain" (C2-P1); "our **manager** is helpful , if she sees we are busy and a bell is going off she will go in to see what is going on, and if the patient is having pain she will then come to notify us" (C2-P8); "I call **APS** when I don't know what else to do for patients with excruciating pain" (C2-P2) ; " we use **Anesthetist** if post op pain" (C2-P5); " In our nursing rounds, I find **OT, PT**, all of the pain services I think, like everybody gets involved" A(AC2-P1, P8); " **Pharmacists** have been really good with helping us too, they spend time on our unit, they go through all the charts, make recommendations" (C2-P8); "others like **health care aids** are helpful" (C2-P4); "Sometimes just having **family** visitors in to distract them, you know like it can decrease their pain because it occupies their mind" (C2-P5,P8). | ID1, ID5, ID6, Rt4, Rt6  , |
|  | Senior nurse as mentor or influencer |  | N=2 C4,C5 | N=8 G1,G2,G3,G4,G5,G6,G7,G8 |  |  | " a lot of the time with new nurses particularly, we'll have patients that wind up in a pain crisis and we try and help each other" (C1-P5)   " Everything I've learned about pain control has come from other nurses " ( C2-P6)."I was trained by senior nurses and the knowledge gets passed and then I think I do it the same way " (C2-P5). |  |
|  | Collaborative pain expert consult/resource |  | N=7 C1,C2,C3,C4,C5,C6,C7 | N=7 G1,G2,G3,G4,G5,G6,G8 |  |  | "We usually consult Advanced Practice Pain Service when we have people who have difficult situations with pain or medicine can’t control their pain themselves, so then they will consult them to come in" (C1- P1). "The APS team is a specialty and I think it’s a really good resource for us" (C1-P6).  "We have the palliative pain care service which is amazing and they teach things that you don’t think about often, like what maybe is causing the pain" C2-P1). | ID5, ID6 |
|  | Workload/  staffing |  |  |  | N=5 C2,C4,C5,C6,C7,C8 | N=7 G1,G2,G3 G4,G6,G7,G8 | " if the floor’s busy, or we are short staffed, this obviously is a barrier" (C1-P6,P8). “I try and keep on top of things. But for sure, it has an influence, I mean you try to get in there and get their pain meds exactly when they need them, but you can’t always. It’s just a reality of our hospital situation" (C1-P7). "Sometimes one nurse for six patients is not enough to maintain and control pain levels" (C1-P5).  "Barriers, I’d say are how busy the unit is, staffing levels, and sometimes we just like can’t get there" (C2-P2, P6). " We have such a quick turnover in patient load that you could have a perfect assignment and within 30 seconds all of a sudden you’ve got three people leaving and three new ones are going to be coming and you don’t know what’s coming through that door next and now hourly rounding becomes every couple hours because you just, you can’t get there in time" (C2-P8). |  |
|  | Frequent MD/Resident rotation changes |  |  |  | N=4 C5,C6,C7,C8 | N=3 G1,G2,G3 | "I mean when (physicians) they’re on board and they’re knowledgeable about the patient it’s all good, but we have a frequent rotation. In between is a difficult time. Things still can be done, but you need to chase people and that’s when things can get a little difficult because you are busy and it’s not just the one patient that is having a new staff, a new resident looking after them, or even student, but rather it’s all of them at once and everybody who has an issue, things have to be dealt and it can take some time and it can take a lot of phone calls, a lot of sitting waiting for answers, so yeah, absolutely. I do enjoy the teaching aspect of the hospital and I think it’s necessary, but sometimes it can be a detriment when trying to get things done" (C1-P7).  "They just changed maybe two weeks ago on my stretch that I had off. I come back; I knew nobody" C2-P2). " You just get them (Residents) the way they like them and then away they go " (C2-P1) |  |
|  | Time management |  |  |  |  | N=4 G3,G5,G7,G8 | "In terms of time, I think the pain will be addressed but it could be done better, the bare minimum will be done, sometimes you just don’t have the time to do the pain assessment" (C2-P7 ). |  |
| supervision | Supportive Leadership – Manager |  | N=7 C2,C3,C4,C5,C6,C7,C8 | N=4 G5,G6,G7,G8 |  |  | "Our manager is very supportive of us doing initiatives trying to improve the general care provided on the unit" (C1-P5).  "my manager is good at encouraging us to go on the education days, and hear about the new meds, new techniques, new ways to control pain" (C2-P6). | ID2, Rt4, Rt5 |
|  | Supportive Nursing Dept Shift Coordinator |  |  | N=1 G7 |  |  | " on nights too if there’s ever a big issue you can call the nursing coordinator for help" (C2-P7). | ID2, Rt4, Rt5 |
|  | Supportive Clinical Care Leader |  | N=6 C1,C2,C3,C4,C5,C8 |  |  |  | "The Pain P/P is always reinforced with our Clinical Care Leaders" (C1-P2) | ID2, Rt4, Rt5 |
| Org culture/climate | Reorganization of wards into 1 - Merged staff & cultures |  |  |  | N=1 C5 |  | "In terms of barriers I would say with the merge between our units that we’ve recently had, the two different Medicine units became one, and then with the change of having less of the long-term care type of patients and having more acute medical need patients, I think it’s taken a back seat to just urgent patient care. So, we’ve had staffing issues, like we just don’t have staff. Also, we were two very different cultures. Two very different approaches to patient management on the unit and it’s been I think about six months now since we merged and things are finally starting to settle, they’re starting to kind of be a meshing of the two cultures, but there was a lot of head butting when it first happened" (C1-P5). | Website, Rt4, Rt5, Rt6 |
|  | Culture of doing research ( Colleague doing research on Pain indicators |  | N=3 C4,C5, C8 |  |  |  | "One of our nurses is doing part of her Master’s project on our unit re: use of non-verbal pain indicators and how that was working on the unit, so that did bring a lot of awareness to it (Pain P/P) when she was going around doing a survey and she did send out an actual tool and she asked people to use it and if this would be helpful and if they found it gave any better indication for pain" (C1-P5). |  |
|  | *Lack of Familiarity/awareness (or lack) with Pain P/P |  |  |  | N=1 C5 | N=3 G4,G5,G6, | "I would say lack of awareness of the Pain Policy, I mean everybody knows it’s supposed to be part of the hourly rounding and that we’re supposed to be collaborating with them and all of that but I don’t think nurses actually read anything on it, it’s was told to you in your orientation and you just kind of integrate into your practice but I don’t think many people refer to it" C1-P5)  "I’ve never seen this pain policy before, I mean all of these things were taught to me but I didn’t know that this was an actual legit document" (C2-P6). |  |
|  | Team culture embraces new initiatives/approaches |  | N=2 C2,C5 | N=5 G3,G4,G5,G6,G8 |  |  | "...our team approach on the unit is very supportive with new initiatives" (C1-P5); "…and open to the use of alternative therapies" (C1-P2).  "our team has a culture that accepts new treatments modalities, I think we're always updating ourselves in terms of pain control, we're using a lot more things like alternative therapies, we have people come in a do acupuncture now, and we are bundling up meds too now for the different kinds of pain, like for neuropathic pain" (C2-P3). |  |
| Training processes | Lack of Pain mgmt resources available |  |  |  |  | N=3 G3,G4,G8, | " a formal guideline or protocol (*clinical pathway*) for pain control would be helpful " (C2-P4); "or pain standing orders" (C2-P3)."we don't really have too many in-services anymore, like on different medications by pharmaceutical companies...also we have limited posters and resources on pain conferences and alternative therapies on the unit, so things like that would be really great" (C2-P8). | ID5, ID6 |
| Information systems= org communication capacity for monitoring (exchange and feedback) | Unrealistic Charting expectations |  |  |  | N=2 C7, C8 | N=7 G1,G2,G4,G5,G6,G7,G8 | "As far as pain goals are concerned, I don’t really think it is established. The pain goals themselves, I mean you’re generally trying to get them comfortable and get them to the point where they can mobilize and have daily activities so they’re not going to be impaired by their immobility. I don’t know of anybody who has actually gotten an actual goal and says ok we’re going to get you to a 3.5 pain score, you know" C1-P7; “the Education Form is probably an unrealistic form. Like again, time. A lot of these policies are made for ideal situations that don’t occur anywhere that I’ve ever worked" C1-P7; "I realize now that I don’t chart my teaching on the use of pain medications but I do document in the teaching when I’m discharging a patient on their pain medications, how often they should take it, when their last one was"(C1-P8).  " I can’t say I’ve ever even written pain goal. Patient states 'wants pain goal to be whatever', I can’t say I’ve ever written that" C2-P2); "we do education all the time, sometimes we don't even know we're educating, but I can't necessarily say I'd write I educated patients regarding pain in this way" (C2-P-2); " you just chart the issues, the problems so if it doesn't present a problem that I have to solve on my shift, I don't think of doing this or take a specific note for that" ( C2-P5); " | ID5, ID6, Rt1(charting practices)    Rt7, ID5, ID6, (Pain Goal)  ID17, Rt7 (Pt Edu) |
|  | EPIC system utility (operating steps ) |  |  |  | N=3 C5,C6,C7 | N-1 G3 | "EPIC is based on a system in the States where you had to record everything because everything made money; this is the system we’re dealing with right now. It doesn’t make it terribly good for patient care but it makes it good for record keeping" C1-P7)  " sometimes the EPIC system can be a big barrier because of all the steps you have to follow, I find it a little frustrating (with EPIC), you’re supposed to be doing this stuff at the bedside but you have to go get your medication out at the OMNI cell,... you have to scan your patient so usually when you’re giving a narcotic or something like that you have to either override scanning the patient or override the med bedside because you can’t do both. to waste medications, it's so frustrating go back an forth from the bedside EPIC system to scan your patient then go to the Omni cell to get med and waste unused part" (C2-P3). |  |
|  | Bedside shift reports |  | N=2 C6,C7 | N= 4 G1,G2,G3,G6 |  |  | "The communication about pain care goes from shift to shift at bedside now, instead of the way we charted before because the new electronic charting system is based on charting by exception" (C1-P6).   " Here at bedside shift report a lot of nurses are fantastic at saying this person has chronic pain or this person’s been having issues with this or that. It’s really useful to know so I don’t have to go searching or ask people"(C2-P2). | Rt3, ID2 |
|  | In room documentation system (Whiteboards) |  | N=1 C8 | N=7 G1,G2,G3,G4,G5,G6,G7 |  |  | "At the bedside, there is a care board (whiteboard) where the patient’s pain level is usually on and the patient will adjust it up and down based on their pain so there’s communication going on between the patient and the nurse all the time about their pain level but it’s not evident on the chart, it’s evident in the room and so the white board is not in any kind of documentation on the patient’s chart" (C1-P8).   "A facilitator I’d say is the white board, it has a pain scale that people will have written in what’s the goal, what’s the base line and it’ll prompt me like oh are we near your goal, or how far off are we? (C2-P6). "I like the whiteboards, when I think about using them I not only just like circling the pain but I’ll actually write on the whiteboard how often they can have pain meds or things like that and that helps to cue the patient and to cue me when I go in there, and the next nurse" (C2-P4). | Rt3, ID2 |
|  | MD & Nurse communication on unit |  |  |  |  | N=3 G2,G3,G8 | "Physicians never prompt a nurse about pain, very rarely. So I feel like if they want to improve maybe the physicians can also prompt nurses and vice versa but it could be that doctors don’t know either and I mean that could be an area of improvement. Physicians don’t really talk or communicate very well with nurses so we have to kind of go and search and find our answer for pain management" (C2-P2); "Senior residents tend to listen to nurses more" (C2-P3). “a few doctors think patients are just drug seeking and don't listen to nurses opinion" (C2-P8). |  |
| business model structure & system to monitor/manage innovation | Collaborative supportive daily interdisciplinary rounds |  |  | N=3 G1,G2,G7 |  |  | “at 2 pm there's nursing rounds where all patient issues come up " (C2-P1). “In daily rounds, we usually discuss barriers to discharge, but if pain management is an issue, we talk about it with the team" (C2-P2). |  |
| NEW | Physical structure/layout |  |  |  | N=2 C1,C4 | N=2 G3,G8 | "We’re a large unit, I think we’re over, we’re almost 80 beds, like we’re, yeah it’s a really large unit" (C1-P1), "and part of unit is on another floor with only 1 RN" (C1-P4).   " Three units make up our unit, AMA , ICU and monitoring "(C2-P3) | ID1, ID2, Rt4, Rt6, Website |
| **DSF Broader System Construct (below)** | | | | | | | | |
| Population characteristic’s | Diverse population needs/  characteristics |  | N=8 C1,C2,C3,C4,C5,C6,C7,C8 | N=6 G2,G4,G5,G6,G7,G8 |  |  | “I think our population has a huge factor because I feel like a lot of people come in with some sort of pain. Many of our patients are multiple core morbidity so either it’s a chronic pain or it’s an acute pain. I feel like Medicine is so diverse" (C1-P1). "...we have a lot of drug abuse patients here and so of course they’re pain management is different"C1-P7).   "it’s acute internal medicine but we’re predominantly a stroke unit, with geriatrics and a patient population with dementia" (C2-P2). | Website, Rt4, Rt5, Rt6 |
|  | Patient beliefs/cooperation |  |  |  | N=4 C2, C4, C7, C8 | N=3 G1, G4,G5 | "one the other things that does impede is certain patients who have already gotten their opioids will just dismiss anything else despite education, and so you don’t even do the Pain Assessment at that point, they’re like “I want it on the hour, when it’s due, on the minute” "(C1-P2).  "they’ll (family) tell you that, Oh my dad or mom has chronic pain, because patients don’t want to bother you and everything" (C2-P2). "...sometimes people are afraid to have pain medication " (C2-P1). |  |
|  | Family beliefs /cooperation |  |  |  | N=4 C2, C4, C7, C8 | N=4 G1, G4, G5, G8 | "If families of patient don´t cooperate can be a barrier" (C1-P6).  "Family or their religion is a factor if they don’t believe in taking medication or they’re scared to ask for it" (C2-P8). "or families are afraid for their loved ones to have pain medication because they don’t want them to be too out of it to be able to talk to them" (C2-P1). |  |
|  | Patient acuity levels |  |  |  | N=3 C5,C6,C7 | N=2 G6,G8 | "We’re just constantly getting new patients that are acutely ill. We don't have enough time now, because we have constant medical issues happening" (C1-P5). "I’ve been doing this for 32 years, but I’ve found in the last year or so, due to changes in patient make-up, we move our ALC patients fairly quickly, so all of our patients on the floor these days are acutely sick and require a lot more attention" (C1-P7).  "So it’s also a Medicine Unit...we’re the Acute Monitoring Area too. We have all the brand-new strokes. We get, we tend to the higher acuity because of the type of staff we have" (C2-P8). |  |

**Key:**

**N= #** total participants per subcase ID = Internal Document

**C1-8**= Subcase 1 participant codes Website = Study site website

**G1-8** = Subcase 2 participant codes Rt = Report

**Supplementary material 5b.** Unit nurses’ KTIs and documents mapped to DSF constructs

| **DSF Constructs**  **Innovation**    **Practice Setting (Context )** | **KTI Code** | | **Subcase 1 (C1)**  **(N=8)**  **C1-P1-8** = Subcase 1 participant codes | **Subcase 2 (C2)**  **(N=8)**  **C2-P1-8** = Subcase 2 participant codes | **Document** |
| --- | --- | --- | --- | --- | --- |
| **DSF Innovation/Intervention Construct (below)** | | | | | |
| Characteristics of innovation | **Routinize recommendations into nursing forms and practices/processes** | | Unit -Embed Prompts integrated into forms (n=5)   - Flowsheets, admission history, brief pain inventory (BPI), medication forms- MAR.     Unit - Embed Prompts integrated into routine practices/processes   - Admission processes (n=5) - Ongoing processes (e.g., shift assessments, hourly rounding, care boards/whiteboards) (n=8) | Unit- Embed Prompts integrated into forms (n=7)   - Flowsheets, admission history, brief pain inventory (BPI), medication forms- MAR.   Unit - Embed Prompts integrated into ‘routine practices/processes’   - Admission processes (n=2) - Ongoing processes (e.g., shift assessments, hourly rounding, care boards/whiteboards) (n=8) | ID2, ID4-6, Rt3, ID12-20 |
| Delivery platform | **Digitalized Pain P/P and forms** | | Dept. - Digitalized policy and forms into new electronic patient information chart (EPIC) (N=6)   - Nursing forms are digitalized with prompts(n=6) - Policy is digitalized | Dept. - Digitalized policy and forms into new electronic patient information chart (EPIC) (N=6)   - Nursing forms are digitalized with prompts(n=6) - Policy is digitalized |  |
|  | | **DSF Practice Setting (Context) Construct (below)** | | |  |
| Staffing =Human resources & capital resources exists within the practice setting | **Engages IP stakeholder involvement** | | Dept.- Pain P/P remains corporate priority and IP policy   - requires all professions to follow (n=3) - IP participate on internal quality committees   Unit- members on pain management strategies (n=3) | Dept.- Pain P/P remains corporate priority and IP policy   - requires all professions to follow (n=3) - IP participate on internal quality committees   Unit - Consult with IP members on pain management strategies i.e. includes pain experts APS, PCS) (n=2)  Unit – Collaborating in daily IP rounds on unit (n=3) C2 –P1,2,7 | ID2, ID3, ID20, Rt3-4, Rt6, |
|  | **Mentorship used by senior nurses to support Pain P/P use** | | Unit –mentorship strategies used by Senior nurses (n=2)   - Senior nurses’ mentor/train novice nurses on pain mgmt. strategies to relieve pain at bedside C1-P4,5 | Unit – mentorship strategies used by senior nurses (n=4)   - Senior staff on the floor really works like a family, they always can help C2-P7 especially in pain crisis C2-P2 (n=2) - Teaching non-verbal language and pt.’s signs C2-P2 and about special pts as Crohns. C2-P2 (n-1) - Novice to senior nurse pain care practice over time changes with experience (n=2) C2-P1,8 - Sharing pain care support/ideas based on experience (n=2) C2-P2,6 |  |
| Org culture/climate | **Fostering an IP and EBP culture among IP team to support Pain P/P use** | |  | Unit – fostering a close, dynamic family style communicating or collaborating on pain issues (n=3)   - very close, family dynamic style of interaction on the unit (n=-1) C2-P8 when communicating and collaborating with each to deal with pain issues (n=3) C2-P3,6,8   Unit – encouraging members to embracing new modalities and improvements (n=5)   - team that accepts/embraces new txmt modalities and ongoing improvements (n=5) C2-P3,4,5,6,8 - Unit - Continuously updating team (n=2) - In terms of pain control using new alternative therapies, and medications. (n=2) C2-P3,6 |  |
| **Information systems= org communication capacity for monitoring (exchange and feedback)** | **Establishing effective communications between providers** | | Unit - reporting practices (n=3)   - Bedside report at shift change to inform patient pain status and how they did with strategies used to manage pain (n=2) C1-P6,7 - communication shift to shift via report and less with documentation now because is based on charting by exception (n=1) C1-P6 - use of whiteboards for communicating pain scores using scales (1-10 and face) (n=2) C1-P7,8 and goal (n=1) C1-P7 - use of clipboard to document pain level in am to charge nurse but not on chart (n=1) C1-P7 | Unit - reporting practices (n=8)   - Bedside reporting - shift to shift - The patient’s status and treatment for pain mgmt. are explained to the next shift Nurse. (n=4) C2-P1,2,3,6 - Use of whiteboards for communicating pain scores (n=3) C2-P1,3,8 and goal (n=5) C2-P2,4,5,6,7 |  |
| supervision | **Leadership strategies** | | Dept.-performance goals related to BPGs in evaluation  Unit - Clinical Care Leaders (n=5)   - get involved in unit wide issues (n=5) C1-P1,2,3,4,5,8 - support ongoing improvements (n=2) C1-P2, 5   Unit- Manager (n=7)   - gets involved to solve unit-wide problem, ones that occur multiple times (n=6) C1-P2,3,4,6,7,8 - provides flow diagram, with statistics about how our unit was doing, with different things to reinforce implementing target behaviours (n=2) C1-P2,4 - Managers use staff meetings are learning forums (n=1) C1-P4 | Dept.-performance goals related to BPGs in evaluation  Unit - Clinical Coordinator (n=1)   - If it is a big issue you can call the nursing coordinator but usually a senior nurse helps (n=1) C2-P7   Unit- KTI Manager (N=4)   - Encourages staff to go on the education days to learn new medication and techniques for pain care and to share at staff meetings (n=1) C2-P6 - reviews incidents and strategies to prevent them in staff meetings (n=1) C2-P5 - Manager is available, helpful and is aware of unit pt.’s needs (n=2) C2-P7,8 | Rt2, Rt4-7, ID7-11 |
| Training processes | **Ongoing Education and Training to support Pain P/P use** | | Dept. – by NPP representatives (n=7)   - General hospital orientation - Pain education days annually offered (n=6) C1-P1,2,3,5,6,7 - Mandatory on line pain education modules (ELM) (n=3) C1-P1,4,5 - In-services on pain for targeted populations (n=3)   (i.e. GPA training to identify what behs mean for pt with dementia C1-P3,4,7)  Unit -provided by Educator (n=7)   - Updates (N=1) C1-P6 - Refreshers on pain assessment (n=2) C1-P4,7 - Refer to Educator to solve when a problem occurs multiple times (n=5) C1-P2,3,6,7,8 - 1 on 1 training (n=3) C1-P1,3,8 - Biannual seminars on pain mgmt. (n=1) C1-P2, | Dept.– by NPP representatives (n=5)   - General hospital orientation - Education days every year (n=5) C2-P2,3,4,6,8   Unit -provided by Educator (n=5)   - Answers 1:1 question (n=2) C2-P2,6 - Refreshers on policy updates or changes (n=3) C2-P4,5,7) - Inservice are available. (n=3) C2-P5,6,8 - nursing staff stroke training (n=1) C2-P2 | Rt2-7, ID1-2 |
| **business model structure & system to monitor/**  **manage innovation** | **Monitoring and evaluation** | | Dept.- ongoing training of surveyors biannually  Unit- audit and feedback (n=2)   - Timely sharing of audit data with unit and seeking remedial action plans (n=2) C1-P1,4 - Manager gets involved in unit wide issues (n=1) C1-P4 - Manager provides flow diagram, with statistics about how our unit was doing, with different survey data compared to other units which are helpful (n=2) C1-P1,4 - Focusing of biannual audit questions to target BPG behaviour | Dept. – ongoing training of surveyors biannually  Unit- audit and feedback (n=1)   - Timely sharing of audit data and seeking remedial action plans (n=1) C2-P5 - Pt satisfaction survey done yearly and shared (n=1) C2-P5 - The exit survey includes pain relieved (n=1) C2-P5 - Manager reviews incidents and strategies to prevent them in staff meetings (n=1) C2-P5 - Focusing of biannual audit question to target BPG behaviour | ID1, ID7-11, Rt1-7 |

**Key**

N=frequency of response by participants BPG – Best practice guideline, APS = Acute Pain Service

Dept.= Department level KTI PT = patient PCS = Palliative Care Service

Unit = Unit level KTI NPP = Nursing Professional Practice GPA =Gentle Persuasion Approach

ID = Internal Document ED = External Document Rt = Report

**References**

1. Chambers DAG, R. E.;Stange, K. C. The dynamic sustainability framework: addressing the paradox of sustainment amid ongoing change. Implementation Science. 2013;8:117.
